# AspC-Mediated Aspartate Metabolism Coordinates the *Escherichia coli* Cell Cycle

**DOI:** 10.1371/journal.pone.0092229

**Published:** 2014-03-26

**Authors:** Feng Liu, Qimuge, Jianfeng Hao, Huijuan Yan, Trond Bach, Lifei Fan, Morigen

**Affiliations:** 1 School of Life Sciences, Inner Mongolia University, Hohhot, China; 2 Department of Pharmacology, Faculty of Medicine, University of Oslo and Oslo University Hospital, Oslo, Norway; University of Massachusetts Medical School, United States of America

## Abstract

**Background:**

The fast-growing bacterial cell cycle consists of at least two independent cycles of chromosome replication and cell division. To ensure proper cell cycles and viability, chromosome replication and cell division must be coordinated. It has been suggested that metabolism could affect the *Escherichia coli* cell cycle, but the idea is still lacking solid evidences.

**Methodology/Principle Findings:**

We found that absence of AspC, an aminotransferase that catalyzes synthesis of aspartate, led to generation of small cells with less origins and slow growth. In contrast, excess AspC was found to exert the opposite effect. Further analysis showed that AspC-mediated aspartate metabolism had a specific effect in the cell cycle, as only extra aspartate of the 20 amino acids triggered production of bigger cells with more origins per cell and faster growth. The amount of DnaA protein per cell was found to be changed in response to the availability of AspC. Depletion of (p)ppGpp by Δ*relA*Δ*spoT* led to a slight delay in initiation of replication, but did not change the replication pattern found in the Δ*aspC* mutant.

**Conclusion/Significances:**

The results suggest that AspC-mediated metabolism of aspartate coordinates the *E. coli* cell cycle through altering the amount of the initiator protein DnaA per cell and the division signal UDP-glucose. Furthermore, AspC sequence conservation suggests similar functions in other organisms.

## Introduction

The cell cycle of slowly growing bacteria is composed of three periods; B, C, and D, and these periods are analogous to the eukaryotic G1, S and M phase, respectively. The B-period represents the time between cell birth and initiation of chromosome replication; the C-period covers the time from initiation to termination of replication; and the D-period is the time between termination of replication and completion of cell division [1], [2]. For a certain strain the lengths of C- and D-periods are fixed (unless the doubling time significantly exceeds 60 min), but that of the B-period depends on the growth rate [3], [4]. When cells grow fast in rich medium the B-period is absent, but the chromosomal replication (C) and cell division (D) periods are detectable. However, still the molecular mechanisms responsible for coordinating chromosome replication with cell division remain unclear.

Initiation of chromosome replication at *oriC* in *E. coli* is finely regulated. The initiator protein, DnaA, exists in two forms, the active form is DnaA-ATP while the inactive form is DnaA-ADP [5]. Binding of DnaA-ATP to low-affinity DnaA-binding sites (I-boxes) in *oriC* leads to unwinding of double-stranded DNA at AT-clusters with assistance of IHF and HU, forming a prepriming open complex [6]. To the open complex, the DNA helicase, the DnaB hexamer, is recruited by DnaC to unwind double-stranded DNA in front of replication forks [7]. After the recruitment of DnaB, the DnaC loader is released and subsequent loading of DNA polymerase III, DnaG primase and SSB assembles two replication forks at one *oriC* and starts replication in opposite directions [8]. Cell division occurs by invagination of the cell membrane at the middle of the cell to form a septum by the FtsZ protein (the Z-ring) that separates the cell into two compartments. FtsZ polymerizes to form a ring structure which sets the site of division and serves as a scaffold for recruitment of other division proteins [9].

It has been suggested that carbon metabolism and fatty acid biosynthesis affect initiation of replication, since mutations in the *pta* and *ackA* gene, which are involved in central carbon metabolism, suppresses the temperature sensitivity of *dnaA*46 [10]. This indicates a link between carbon metabolism and DnaA. Furthermore, absence of YgfZ, a folate-binding protein involved in one-carbon metabolism [11] suppresses the *hda* mutation [12]. YgfZ may therefore be involved in regulation of DnaA-ATP hydrolysis. Mutations of *pgk*, *pgm*, *eno* and *pykA,* whose gene products are involved in glucose metabolism, suppress the temperature sensitivity of *dnaE* [13], suggesting that sugar metabolism may affect elongation of replication. A recent study showed that absence of the FabH protein, which participates in fatty acid biosynthesis, led to production of small cells, indicating that fatty acid biosynthesis plays a central role in regulating the size of *E. coli* cells in response to nutrient availability [14]. Thus, there is considerable evidence to link general metabolism to cell size and therefore indirectly to cell-cycle regulation. Cells grown in rich medium are bigger with more origins per cell than cells grown in poor medium [15]. Hence, cell size has been proposed to be a trigger for initiation of replication [16], [17]. The initiation mass, the cell mass per origin at the time of initiation, is suggested to be constant [18]. However, Wold *et al*. [15] showed that the initiation mass was dependent on growth rate, indicating that timing of initiation of replication is not regulated by a direct connection between mass accumulation and the factors regulating initiation of replication.

In *E. coli*, amino acid biosynthesis and sugar metabolism are closely connected (Figure S1). The AspC protein can synthesize aspartate from oxaloacetate by transferring an amino group and a reversal leads to degradation of aspartate to oxaloacetate (Figure S1). Aspartate is a precursor of different metabolites including amino acids, vitamin B5, NAD, mDAP, and nucleotides. In a global transcriptional analysis, we found that expression of the *aspC* gene was increased in the *dnaA*345 mutant defective for ATP-binding [19], suggesting that DnaA may regulate the expression of *aspC* (Morigen & Skarstad, unpublished data). This connection between DnaA and the *aspC* gene led us to investigate the role of AspC in control of the *E. coli* cell cyle.

We found that the *aspC* mutant cells were smaller with fewer replication origins and had an increased doubling time. Excess AspC had the opposite effect. Since this study shows that AspC function is crucial in coordination of the cell cycle, we propose that AspC-mediated aspartate metabolism has a key role in coordinating chromosome replication and cell division with cell growth in *E. coli*.

## Materials and Methods

### Bacterial Strains and Plasmids

All bacterial strains used were K12 listed in Table 1. The *aspC::cam* ^R^ or *aspC::kan* ^R^ allele was P1 transduced into *dnaA*46, *dnaB*252, *dnaC*2, *tyrB*, *relAspoT* mutants respectively as described previously [20]. For construction of a triple mutant *ilvEtyrBaspC*, the *kan* ^R^ gene was removed from *tyrB::kan^R^aspC* using the method described previously [22], and then *ilvE::kan^R^* allele was P1 transduced into *tyrBaspC* mutant. PCR fragment of the *aspC* gene with its native promoter region using a pair of primers of 5′ CCCAAGCTTCACCGTTGCTGTGGGTATCGT 3′ and 5′ TCCCCCGGGGCTTTTCAGCGGGCTTCATTG 3′ was inserted into plasmid pACYC177 at HindIII and SmaI sites, resulting in a plasmid over-expresssing AspC, namely pACYC177-*aspC*. The plasmid was introduced into competent cells by eletroporation.

**Table 1:** Bacterial strains used.

Strain	Relevant genotype	Source or Reference
BW25113	Wild type *rrnB3* Δ*lacZ4787 hsdR514* Δ *(araBAD)567*Δ *(rhaBAD)568 rph-1*	Baba *et al*. (2006)
MOR814	BW25113 *aspC::Cam^R^*	This work
MOR816	BW25113/pACYC177*-aspC*	This work
MOR817	BW25113/pACYC177	This work
MOR346	BW25113 *tyrB::Kan^R^*	Baba *et al*. (2006)
MOR820	BW25113 *aspC::CamR tyrB::Kan^R^*	This work
MOR823	*dnaA46(Ts), aspC::Kan^R^*	This work
MOR824	*dnaB252(Ts), aspC::Kan^R^*	This work
MOR825	*dnaC2(Ts), aspC::KanR*	This work
MOR826	MOR814/pACYC177-*aspC*	This work
MOR827	MOR814/pACYC177	This work
MG1655	Wild type *rph-1*	Baba *et al*. (2006)
CF1961	MG1655 Δ*relA* Δ*spoT*	Xiao *et al*. (1991)
MOR828	MG1655 *aspC::Kan^R^*	This work
MOR829	MG1655 Δ*relA* Δ*spoT aspC::Kan^R^*	This work
MOR832	*dnaA46(Ts)/*pACYC177*-aspC*	This work
MOR833	*dnaB252(Ts)/*pACYC177*-aspC*	This work
MOR834	*dnaC2(Ts)/*pACYC177*-aspC*	This work
MOR1618	BW25113 *aspC::Cam^R^ tyrB ilvE::kan^R^*	This work
MOR1344	BW25113 *glyA::Kan^R^*	Baba *et al*. (2006)
MOR1345	BW25113 *ilvE::Kan^R^*	Baba *et al*. (2006)
MOR1346	BW25113 *avtA::Kan^R^*	Baba *et al*. (2006)
MOR1347	BW25113 *dadX::Kan^R^*	Baba *et al*. (2006)
MOR1348	BW25113 *argH::Kan^R^*	Baba *et al*. (2006)

### Growth Media and Condition

Cells were grown in LB, ABTGcasa medium [21], ABTG which was lacking casamino acids (CAA) compared to ABTGcasa medium and ABT medium which was AB salt supplemented with thiamin (10 μg/mL), Ala (0.2%), Met (20 μg/mL), Trp (20 μg/mL), His (20 μg/mL) at 37°C. 50 μg/ml of kanamycin, 30 μg/ml of chloramphenicol and 50 μg/ml of ampicillin were added when required for selection.

### One-step Inactivation of *aspC*

To replace the *aspC* gene by *cam* ^R^ gene, we used one-step inactivation method as described previously [22]. First, a PCR fragment of the *cam* ^R^ gene which bears 50 bp of homologous sequences targeting two margins of the *aspC* gene was amplified using primers of 5′ ATGTTTGAGAACATTACCGCCGCTCCTGCCGACCCGATTCTGGGCCTGGCGTGTAGGCTGGAGCTGCTTC 3′ and 5′ TTACAGCACTGCCACAATCGCTT CGCACAGCGGAGCCATGTTATCTGGTGCATATGAATATCCTCCTTAG 3′ with pKD3 as a template. Secondly, a wild type strain BW25113 expressing Red recombination system from pKD46 under induction of L-arabinose (10 mmol/L) was transformed with the PCR fragment of *cam* ^R^ gene to replace *aspC* by homologous recombination. The resulted transformants were selected on LB plate with chloramphenicol. Plasmid pKD46 which is temperature sensitive was removed by growing the transformants at 42°C. The resulted *aspC::cam* ^R^ mutant was confirmed by PCR and flow cytometry analysis.

### Flow Cytometry Analysis

Exponentially growing cells in LB, ABTGcasa, ABTG medium were collected at OD_600/450_ = 0.15, and then treated with 300 μg/mL of rifampicin and 10 μg/mL of cephalexin for 3–5 generations. Rifampicin inhibits initiation of replication through preventing transcription which is required for replication initiation but allows ongoing rounds of replication to finish, whereas cephalexin blocks cell division [23], [24]. After treatment with these drugs, cells end up with an integral number of chromosomes [23], representing the number of origins per cell at the time of drug addition. Cells treated with rifampicin and cephalexin were fixed in 70% ethanol. Slowly growing cells in poor medium of ABT were collected by centrifugation at OD_450_ = 0.15 and fixed in 70% ethanol. Following one wash in Tris-HCl buffer (pH7.5), cells were stained in Hoechst33258 for 30 min, and analyzed by flow cytometer (BD LSRFortessa). Preparation of standard sample and post analysis were as described previously [15], [21].

### Measurement of Cell Size

Exponentially growing cells in LB, ABTGcasa, ABTG or ABT medium were harvested at OD_600/450_ = 0.15 and fixed in 70% ethanol. Cells were observed and pictured with an Axio Imager A2 microscope (Zeiss) and AxioCam MRC5 camera. Measurement of cell size was then performed based on observation.

### Determination of Total Protein per Cell

Exponentially growing cells in ABTGcasa at 37°C were collected on ice. 9 mL of the cell culture were harvested by centrifugation at 4°C, washed in 1 ml of TE buffer, and resuspended in 200 μl TE buffer containing 1% SDS and glycerol and then boiled for 5 min. Total protein amount in a fixed volume of the cell extract mentioned above was determined by a colorimetric assay (BCA kit, pierce) as described previously [25]. To measure the number of cells in a certain volume of the cell culture, 10 μL of the cells mentioned above were diluted 10^4^ and 10^5^ times and then plated on LB agar plates with required antibiotics. After incubation at 37°C overnight, the number of the colonies were counted. Using the amount of protein in a certain volume of cell extract from 9 ml of the culture and the number of cells in 10 μL of the culture, the protein amount per cell was calculated.

### Western Blotting

The cell extract mentioned above was also used to determine the DnaA concentration by Western blotting. Fixed amounts of cell extracts were subjected to SDS-PAGE (12%). The protein was transferred to a polywinylidene difluoride (PVDF) membrane by semi-dry blotting. The membrane was probed with anti-rabbit antibody for DnaA and secondary antibody which was also anti-rabbit IgG (TransGen Biotec) as described previously [26].

## Results

### Deletion of the *aspC* Gene Decreases the Number of Origins per Cell

**Figure 1:**
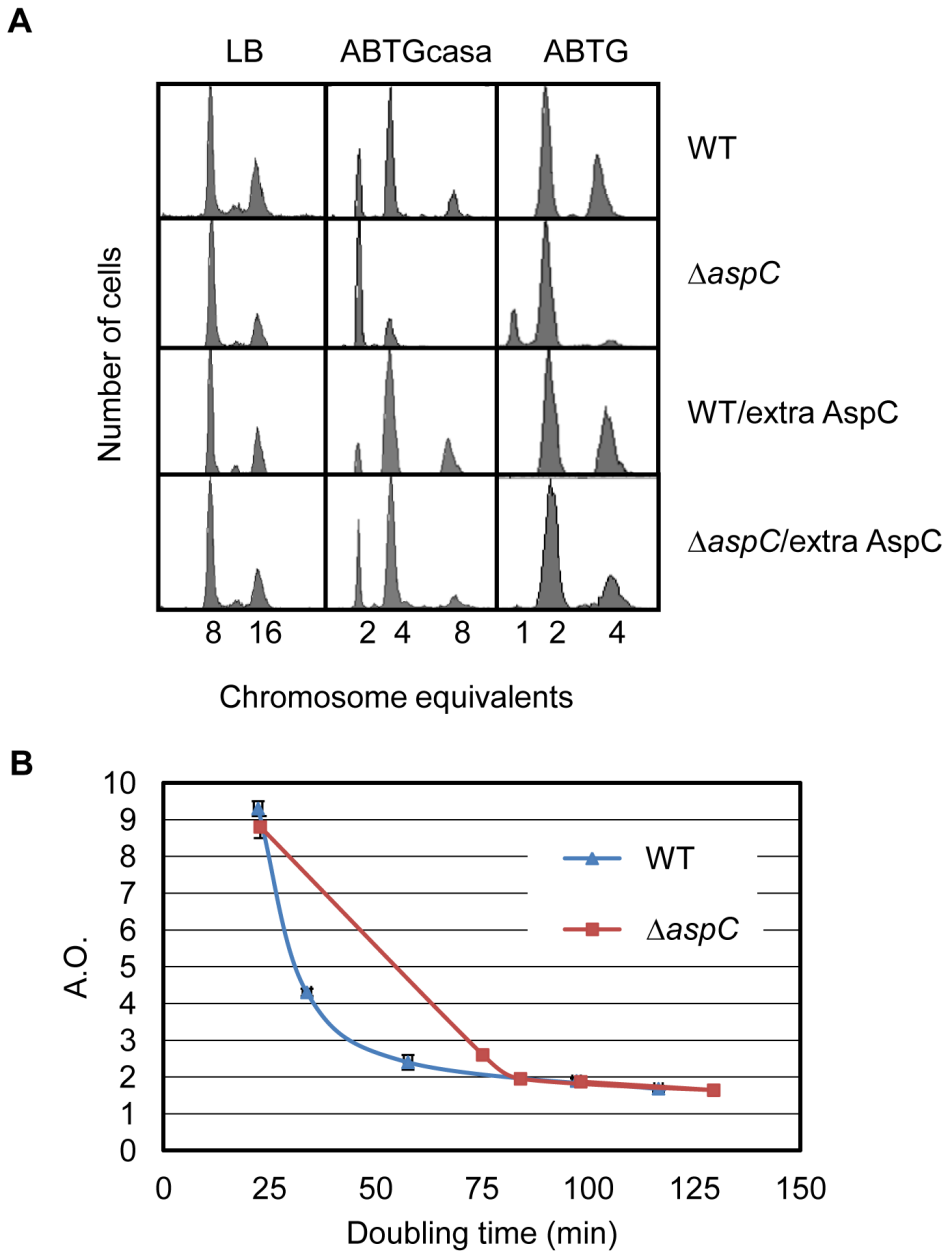
Deletion of the *aspC* gene decreases the number of origins per cell and the decrease is reversed by extra AspC. (A) Exponentially growing cells in LB, ABTGcasa or ABTG medium at 37°C were treated with rifampicin and cephalexin for 3–5 generations. The number of fully replicated chromosomes per cell represents the number of origins present at the time of drug addition. Subsequently, cells were fixed in 70% ethanol and analyzed by flow cytometry as described in Materials and Methods. For each analysis, 10000 cells were included. Extra AspC was produced from the plasmid pACYC177-*aspC* in which the *aspC* gene is under control of its native promoter. (B) The number of origins per cell in exponentially growing cells in LB, ABTGcasa, ABTG, ABT with aspartate or ABT medium at 37°C was measured as described in A. The average number of origins per cell was plotted as a function of the doubling times. Data points are average of three individual experiments and standard errors are given as error bars.

To moniter the replication pattern of the Δ*aspC* mutant, exponentially growing cells in ABTGcasa medium (see Materials and Methods) at 37°C were treated with rifampicin and cephalexin for 3–5 generations, and then analyzed by flow cytometry. Rifampicin blocks initiation of replication, but allows ongoing elongation to finish [23], whereas cephalexin stops cell division [24]. Wild-type cells contained 2, 4 or 8 origins, with an average of 4.3 origins per cell, while the Δ*aspC* mutant cells contained 2 or 4 origins with an average of only 2.6 origins per cell (Figure 1A). Therefore, the average number of origins per cell was decreased by 40% in Δ*aspC* mutant relative to wild type. The doubling time of wild-type was 34 min and that of Δ*aspC* was 75 min, showing that growth of Δ*aspC* was severely slowed down. The cells were also grown in ABTG medium without casamino acids at 37°C. Flow cytometry analysis showed that wild-type cells, with doubling time of 58 min, had 2 or 4 origins, whereas the Δ*aspC* mutant cells, with a doubling time of 84 min, had 1 and 2 origins (Figure 1A, Table 2). The average number of origin per cell was decreased from 2.4 in wild type to 2.0 in Δ*aspC* cell (Table 2).

**Table 2:** AspC-mediated metabolism specially affects cell cycle parameters.

strain	genotype	A.O.	Cell size(μm)	Doubling time(min)
BW25113	Wild type	2.43±0.19	2.32±0.01	57.6±0.1
MOR814	Δ*aspC*	1.95±0.15	1.83±0.14	84.1±1.9
MOR346	Δ*tyrB*	2.40±0.12	2.36±0.14	67.8±0.6
MOR1344	Δ*glyA*	2.56±0.06	2.21±0.13	53.7±0.6
MOR1345	Δ*ilvE*	2.44±0.01	2.32±0.16	60.3±1.0
MOR1346	Δ*avtA*	2.65±0.01	2.22±0.14	58.7±1.1
MOR1347	Δ*dadX*	2.44±0.15	2.12±0.16	54.9±1.4
MOR1348	Δ*argH*	2.47±0.04	2.31±0.15	59.3±1.0

Exponentially growing cells in ABTG medium (AB minimal salts supplemented with thiamin and glucose) were treated with rifampicin and cephalexin, fixed in 70% ethanol and then analyzed by flow cytometry as described in Materials and Methods. Average number of origins per cell (A. O.) was calculated using a software provided by BD and cell size of exponentially growing cells in the medium above was determined by fluorescent microscopy. Each calculation includes more than 100 cells. The values are average of three individual experiments and standard errors are given.

When Δ*aspC* cells were grown in LB, the decrease in number of origins per cell was not as severe as that observed in ABTGcasa. Wild-type strain had 8 or 16 origins, with an average of 9.3 origins per cell whereas the Δ*aspC* mutant had an average of 8.8 origins per cell (Figure 1A). The doubling time of wild–type cells was 22 min and that of the Δ*aspC* mutant was 23 min.

When cells were grown in the poor medium ABT (see Materials and Methods) at 37°C, differences in cell cycle parameters were also observed in Δ*aspC* mutant relative to wild-type cells (Table 3). Fewer cells in the C period and more cells in the B period were found for the Δ*aspC* mutant compared to wild-type cells, but the growth rate of the wild-type and Δ*aspC* mutant were similar (Table 3). These results suggest that absence of AspC delays initiation of replication.

**Table 3:** Additional aspartate leads to a production of bigger cells and more C-period cells.

Strain	genotype	a.a.	B(%)	C(%)	D(%)	Cell size(μm)	Doubling time(min)
BW25113	Wild type	–	39.6	39.7	20.6	2.09±0.14	116.6±0.2
		Asp	29.0	47.2	23.8	2.29±0.13	87.3±1.2
		Gly	34.7	44.0	21.3	2.04±0.13	99.7±0.3
		Tyr	38.4	41.4	20.2	2.04±0.16	114.5±0.8
MOR814	Δ*aspC*	–	46.0	34.9	19.1	1.80±0.14	129.6±0.8
		Asp	30.5	45.0	24.5	2.16±0.15	98.3±0.5
		Gly	45.5	36.4	18.1	1.86±0.22	121.2±1.7
		Tyr	68.8	19.3	11.9	1.76±0.12	430.9±3.5

Exponentially growing cells in ABT medium supplemented with amino acids as indicated were fixed in 70% ethanoland then analyzed by flow cytometry as described in Materials and Methods. The number of cells in B-, C- and D-period was calculated using a software provided by BD and cell size was determined by fluorescent microscopy. Each calculation includes more than 100 cells. The values are average of three individual experiments, standard errors are given.

To see the correlation between initiation frequency of replication and growth rate, we plotted the average number of origins per cell as a function of doubling times. As shown in Figure 1B, the number of origins per cell was correlated with growth rate, but not linearly. Both wild-type and Δ*aspC* cells had 2.4 origins per cell with very different growth rates (Figure 1B). The results suggest that growth rate does not necessarily control the initiation frequency of replication.

### Overproduction of AspC Reverses the Decreaed Number of Origins per Cell in Δ*aspC* Mutant

To examine if overexpression of AspC reverses the phenotype of Δ*aspC*, an AspC-overproducing plasmid was constructed by inserting the *aspC* gene with its native promoter into pACYC177 (a plasmid with moderate copy number [26]). The resulting plasmid named pACYC177-*aspC* was introduced into the *aspC* mutant and its wild-type counterpart. Cells were exponentially grown in ABTGcasa medium at 37°C and the cells were analysed with flow cytometry as described above. When AspC was produced from pACYC177-*aspC* plasmid in the Δ*aspC* mutant, the replication pattern in the Δ*aspC* mutant was reversed to a wild-type pattern with 2-, 4- and 8-origin cells. The 8-origin cells were not found in Δ*aspC* mutant lacking AspC (Figure 1A). The average number of origin per cell increased from 2.4 to 3.4 in response to overproduction of AspC. When the cells were grown in ABTG medium the decrease in the number of origins per cell in the Δ*aspC* mutant was also reversed by excess AspC (Figure 1A). However, the effect of extra AspC was not remarkable when the cells were grown in LB medium (Figure 1A). When excess AspC was expressed in wild type cells the number of 8-origin cells slightly increased, with a concomitant small decrease in number of 2-origin cells relative to the control (Figure 1A, ABTGcasa medium). This shows that overexpression of AspC increases the number of origins per cell and we propose that AspC is a positive factor for initiation of replication either directly or indirectly.

### AspC Affects Cell Size

**Figure 2:**
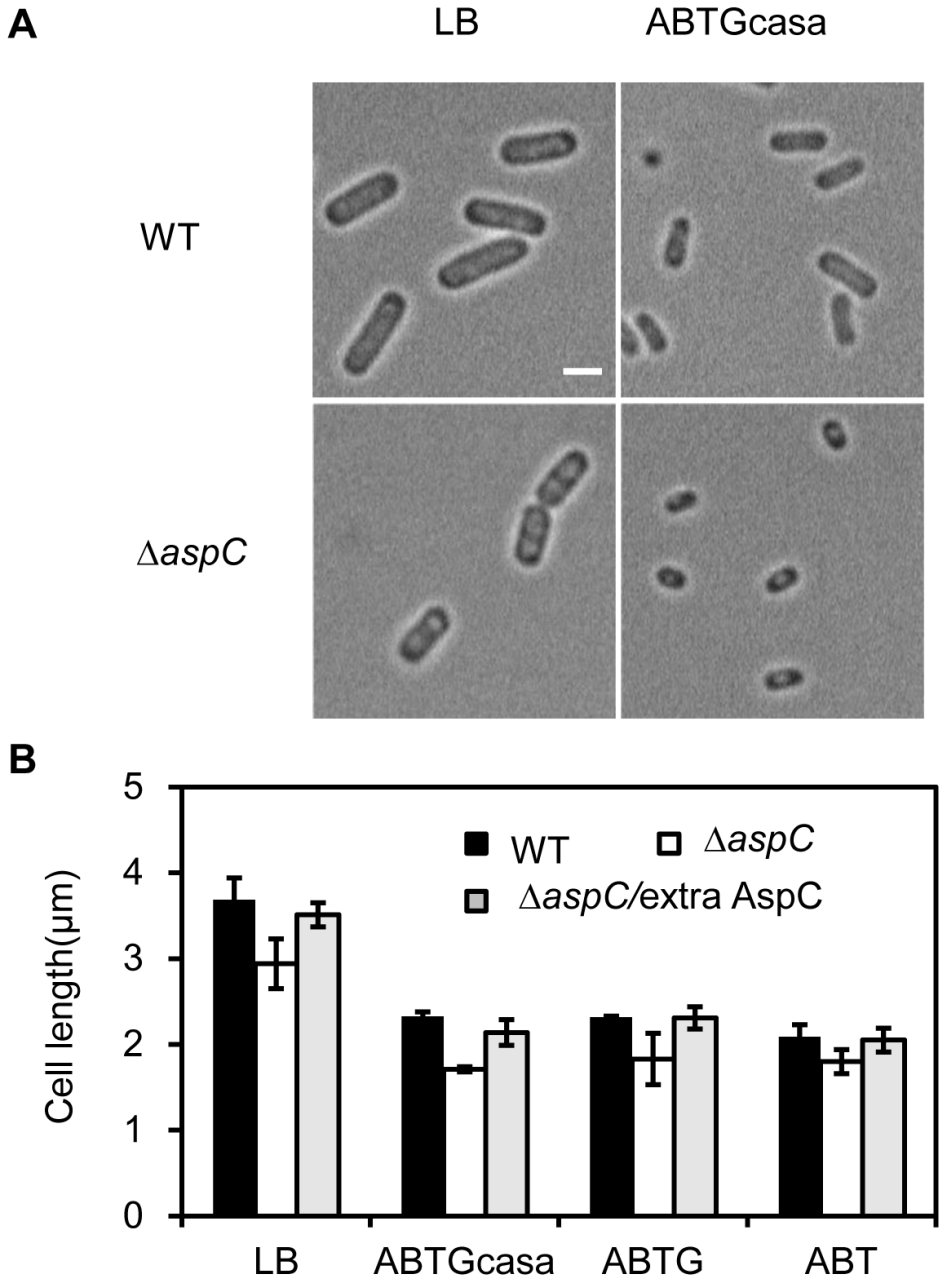
Deletion of the *aspC* gene reduces cell sizes. (A) Exponentially growing cells in LB or ABTGcasa medium at 37°C were visualised by fluorecence microscopy. (B) Cell length varies with nutrient availability. Bar graphs show average cell lengths in different growth media. Cells in LB, ABTGcasa, ABTG or ABT medium at 37°C and the average size of the cells were measured. More than 100 cells were included in each calculation. The values are average of three individual experiments and standard errors are given as error bars.

We have shown that absence of AspC decreases the number of origins per cell and excess AspC does the opposite, indicating that AspC increases initiation frequency of replicatin. To investigate whether AspC also affects cell division we measured cell size in the absence of AspC or in the presence of excess AspC. Exponentially growing cells at 37°C in LB, ABTGcasa, ABTG or ABT medium were collected at OD_600/450_ = 0.15, and fixed in 70% ethanol. Cells were observed using an Axio Imager A2 fluorescence microscope (Zeiss) and cell size was measured. We found that the length of Δ*aspC* cells grown in LB was 2.9 μm compared to 3.7 μm of the wild-type cells. This equals a 21% decrease in cell size (Figure 2) and indicates that absence of AspC not only causes a decrease in number of origins per cell, but also a reduction of cell length. Interestingly, the width of Δa*spC* cells was not found to be changed relative to wild-type cells. When cell length was plotted as a function of nutrient availability (LB, ABTGcasa, ABTG and ABT medium), Δ*aspC* cells were always shorter than wild-type cells. It should be noted that the size of wild-type cells was about 2.1–2.3 μm in ABTGcasa with a doubling time of 33 min (in ABTG the doubling time was 58 min and in ABT 116 min). The size of Δ*aspC* cells was 1.7–1.8 μm in the media mentioned above and with a very different growth rate (Figure 2B, Tables 2 and 3). The Δ*aspC* cells grown in ABTGcasa were smaller than when grown in ABTG or ABT medium (Figure 2B), although the latter two media are poorer than ABTGcasa.

Expression of AspC from the pACYC177-*aspC* plasmid reversed the small-cell phenotype of the Δ*aspC* mutant grown in all media described above (Figure 2B). A slight increase in size of the wild-type cells in the presence of excess AspC was also found in the media used (data not shown). Based on the results described above we conclude that AspC affects cell division either directly or indirectly.

### The Correlation between Initiation Mass and Growth Rate

The initiation mass, the cell mass per origin at the time of initiation, is important for timing of initiation of replication [18], and is dependent on growth rate [15]. When the initiation mass was plotted as a function of growth rate, in agreement with a previous study [15], we found that the initiation mass depended on growth rate in wild-type cells (Figure S2). However, the wild-type and Δ*aspC* cells grown in ABTGcasa medium showed similar initiation masses with very different growth rates. In contrast Δ*aspC* cells had different initiation masses with similar growth rates in ABTGcasa and ABTG (Figure S2). These results suggest that the initiation mass is not directly determined by growth rate.

### The TyrB Protein, Another Aminotransferase, does not Affect Progression of the Cell Cycle

**Figure 3:**
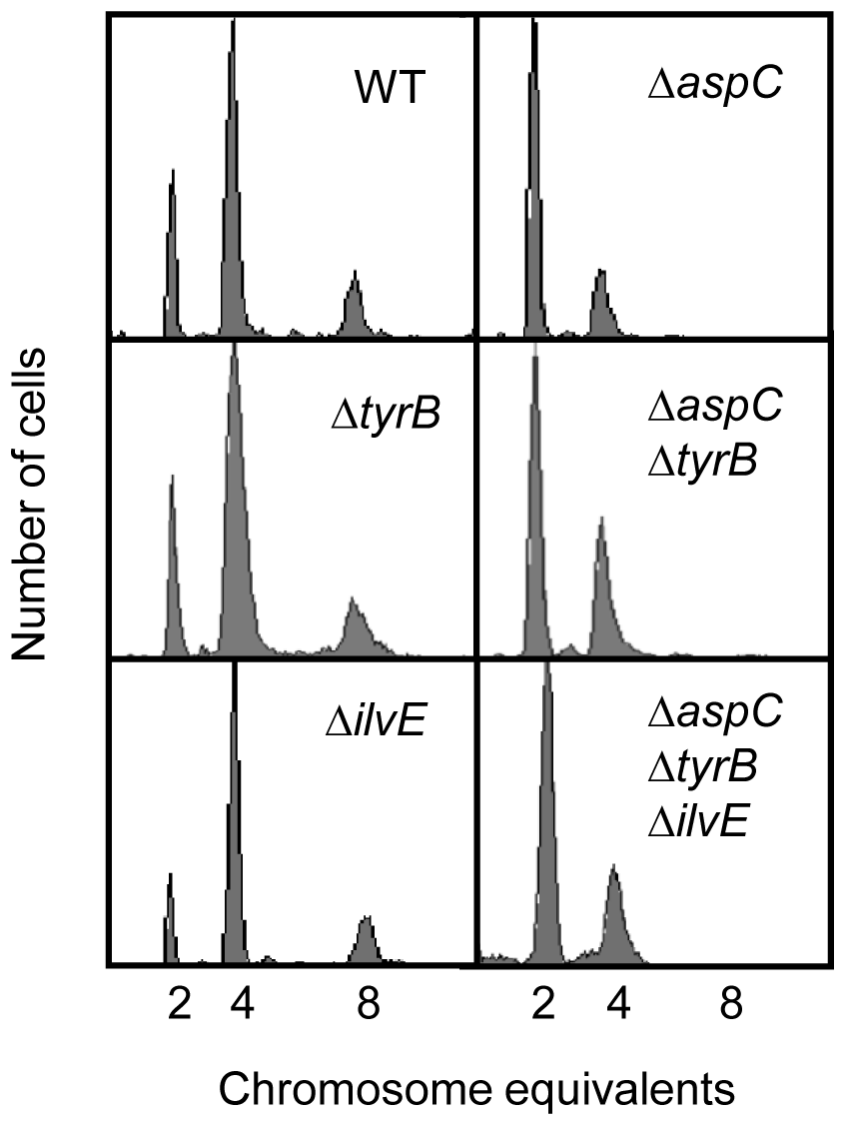
TyrB, an aminotransferase, does not affect initiation of replication. Exponentially growing cells in ABTGcasa at 37°C were treated with rifampicin and cephalexin for 3–5 generations and then analyzed by flow cytometry as described in the legend to Figure 1.

Another aminotransferase TyrB also has the capability to synthesize aspartate, but TyrB is more likely to use aromatic substrates such as phenylalanine and tyrosine compared to AspC which uses oxaloacetate [27]. To check if the effect of AspC on the cell cycle is a specific or a general effect of lacking aspartate, we examined the replication pattern of the Δ*tyrB* mutant in which aspartate synthesis is reduced due to absence of TyrB. In contrast to Δ*aspC*, chromosome replication pattern and cell size of Δ*tyrB* mutant were similar to wild-type in both ABTGcasa and ABTG medium (Figure 3, Table 2). It should be noted that the doubling time of Δ*tyrB* cells was increased 15% compared to that of wild-type cells (Table 2). The Δ*tyrB*Δ*aspC* double and Δ*ilvE*Δ*tyrB*Δ*aspC* triple mutant showed the same replication pattern (Figure 3), similar growth rate and cell size as Δ*aspC*, further supporting that the TyrB pathway of aspartate synthesis does not affect initiation of replication or cell division.

### Defective Synthesis of Other Amino Acids does not Influence the Number of Origins per Cell and Cell Size

**Figure 4:**
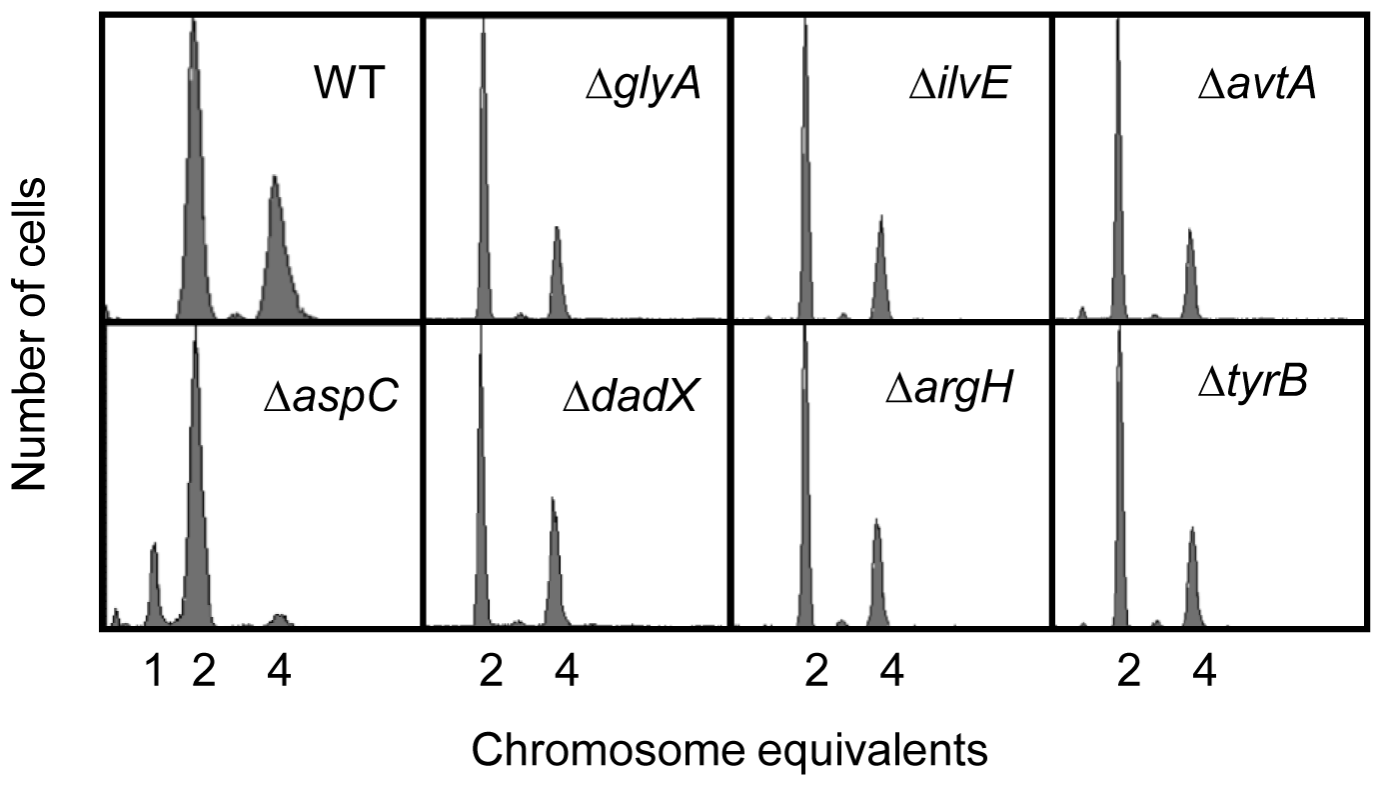
Absence of the AspC protein specially decreases the number of origins per cell. Exponentially growing cells in ABTG at 37°C were treated with rifampicin and cephalexin for 3–5 generations and then analyzed by flow cytometry as described in the legend to Figure 1.

The above data indicate that the absence of AspC protein delays progression of the cell cycle. To examine if the AspC-mediated coordination of the cell cycle is specific we determined the replication pattern, cell size and growth rate of *glyA*, *ilvE*, *avtA*, *dadX*, *argH* and *aspC* mutant cells. The GlyA, IlvE, AvtA, DadX and ArgH proteins are involved in different pathways of amino acid metabolism. GlyA converts serine to glycine, transferring a methyl group to tetrahydrofolate, thus forming 5,10-methylene-tetrahydrofolate (5,10-mTHF). 5,10-mTHF is the major source of C1 units in the cell [28], and thus GlyA is a key enzyme in the biosynthesis of purines, thymidine, methionine. IlvE carries out the final step in valine, leucine and isoleucine biosyntheses [29]; AvtA catalyzes the transamination reaction that converts α-keto-isovalerate to valine and alanine to pyruvate [29]; DadX catalyzes the interconversion of D- and L-alanine [30]; and ArgH catalyzes the final step in the L-arginine biosynthesis [31]. Therefore, the mutants mentioned above are expected to have serious and different defects in intermediary metabolism. Interestingly, the *glyA*, *ilvE*, *avtA*, *dadX* and *argH* mutants grown in ABTG at 37°C showed a wild-type replication pattern with normal cell size and growth rate. In contrast, smaller cells with less origins and slower growth were found in the Δ*aspC* culture (Figure 4, Table 2). This indicates that disruption of biosynthesis of glycine, valine, leucine, isoleucine, alanine, arginine and their upstream precursors do not affect normal cell–cycle coordination, in contrast to that of aspartate. These results suggest that AspC-mediated aspartate metabolism exerts a specific control on the progression of the cell cycle either directly or indirectly.

### Aspartate Stimulates Progression of the Cell Cycle

**Figure 5:**
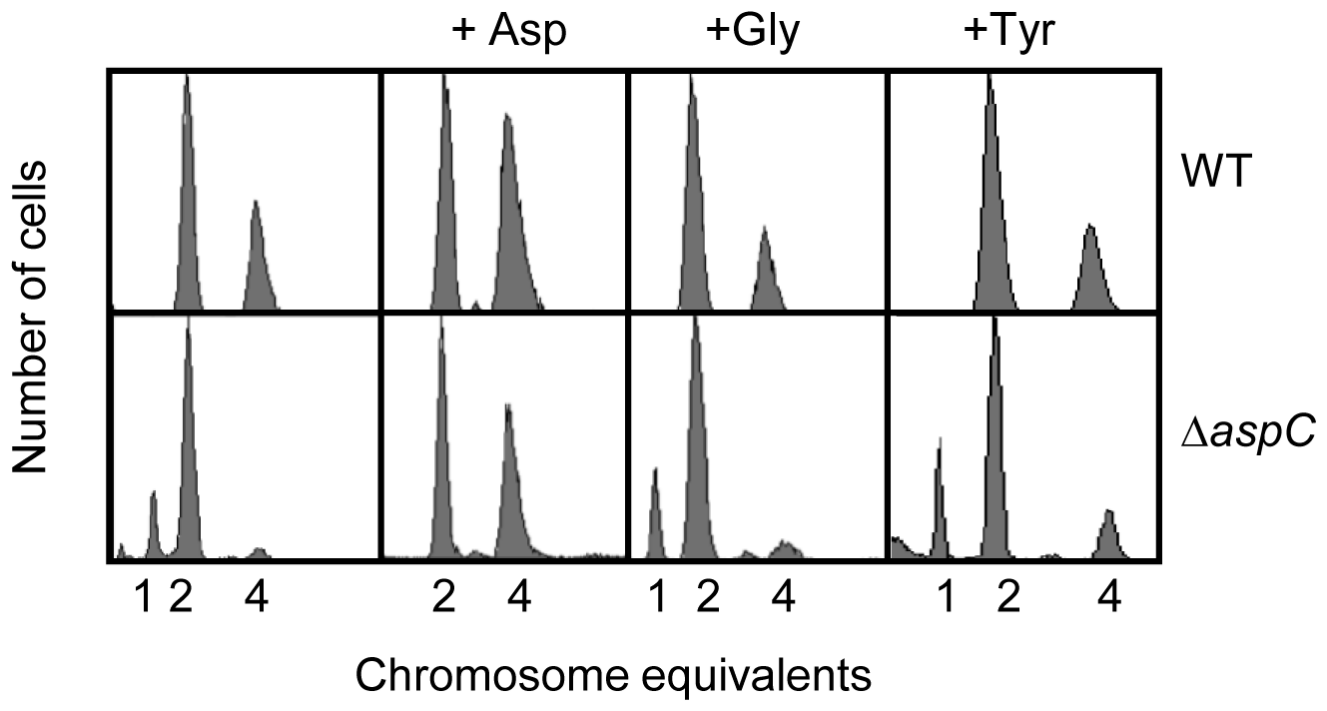
Aspartate stimulates initiation of replication. Exponentially growing wild type or Δ*aspC* cells in ABTG medium at 37°C were supplemented with 100 μg/mL of Asp, Gly or Tyr. Cells were then treated with rifampicin and cephalexin for 3–5 generations and analyzed by flow cytometry as described in the legend to Figure 1.

As mentioned in the introduction, AspC synthesizes aspartate from oxaloacetate by transferring an amino group (Figure S1). To test if the AspC-effect on cell-cycle progression is exerted by aspartate availability, we measured cell-cycle parameters in ABTG medium (see Materials and Methods) supplemented with 100 μg/ml of aspartate or other amino acids by flow cytometry and microscopy. Exponentially growing cells in ABTG medium with supplement of amino acids were treated with rifampicin and cephalexin for more than 3–5 generations, and then analyzed by flow cytometry. Addition of aspartate in the medium increased the number of origins per cell in both wild type and the Δ*aspC* mutant, compared to medium without aspartate. Addition of other amino acids did not change the replication pattern (Figure 5, S3). Concomitantly, cell size and growth rate were also increased in the precence of extra aspartate (Table S1 in File S1). It should be noted that addition of glutamic acid also promoted number of origins per cell and growth rate of wild-type cells (Figure S3), but the cell size was not increased (Table S1 in File S1). The results indicate that of the 20 amino acids, only aspartate affects both replication and division cycles. However, addition of cysteine, alanine or leucine led to a delay of initiation of replication (Figure S3). The negative effect of cysteine on initiation was dramatic (Figure S3), accompanied with great reduction in growth rate and cell size (Table S1 in File S1). The cysteine-effect on the cell cycle may be explained by an imbalance in the concentration of amino acids. When too much cysteine is available in a cell, it may interrupt proper incorporation of other amino acids in protein synthesis. As a result, such an interruption may slow down the progression of the cell cycle.

When growing exponentially in ABT medium (poorer medium) the Δ*aspC* mutant culture showed 46% cells in B, 35% in C, and 19% in the D period, whereas a supplement of aspartate led to 29% cells in B, 47% in C, and 24% in the D period. This shows that addition of aspartate increases the number of cells in C-period and decreases the number of B-period cells (Table 3, Figure S4). The result suggests that aspartate increases initiation frequencey of replication in the Δ*aspC* cells. In accordance with the advanced replication cycle, a remarkable increase in cell size with shortened doubling time was also observed when the extra aspartate was given in the ABT medium (Table 3). However, extra glycine, tyrosine (Table 3), as well as all other amino acids (Figure S4) was not able to recover the delayed cell cycle of the Δ*aspC* mutant. When wild-type cells was grown in ABT medium supplemented with aspartate, both the number of cells in C-period and the cell size were increased and the growth rate was faster relative to control (Table 3). This indicates that aspartate affects the cell cycle specifically.

However, supplement of tyrosine and valine dramatically accumulated the B-period cells (69% and 64% relative to 46% of control) and decreased the growth rate (430 and 246 min relative to 129 min of control) in Δ*aspC* cells. But the extra tyrosine did not have the same negative effects on the cell cycle of wild-type cells (Table 3). This may be explained by an imbalance in concentration of aspartate and tyrosine in the Δ*aspC* mutant since tyrosine is used in a low frequency in protein synthesis.

### AspC Affects the Amount of DnaA per Cell but not the Concentration

**Figure 6:**
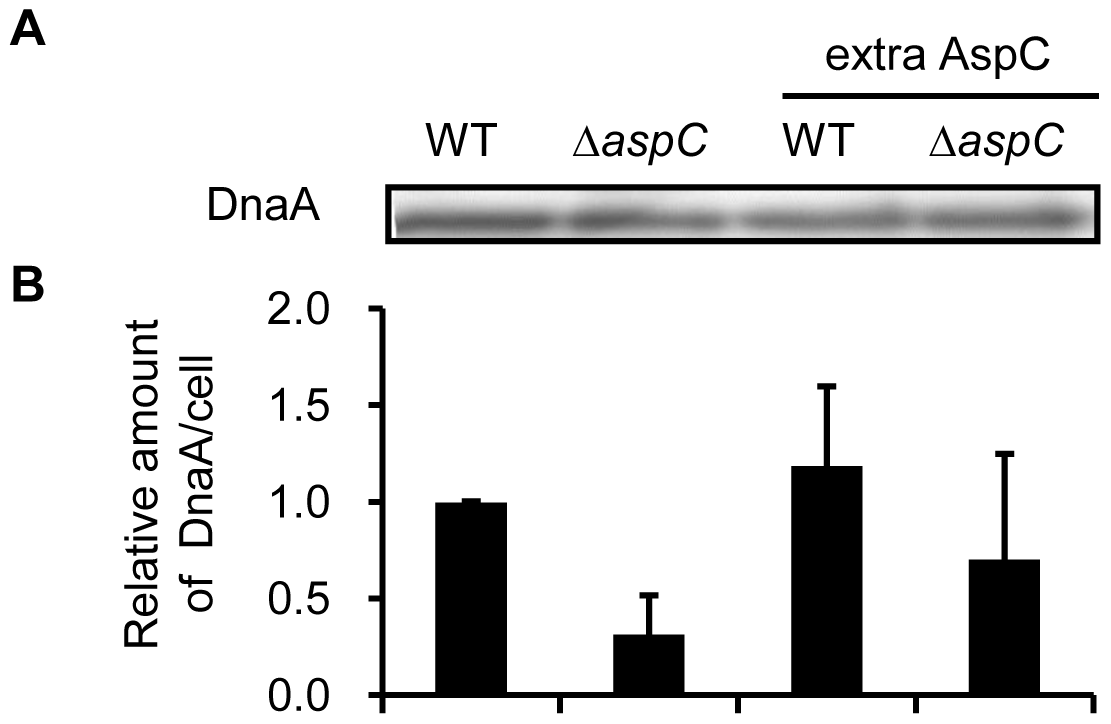
AspC changes the amount of DnaA protein per cell. Exponentially growing cells in ABTGcasa at 37°C were harvested by centrifugation at 4°C. Total protein amount in a fixed volume of cell extract was determined by a colorimetric assay (BCA kit) as described in the Materials and Methods. The DnaA concentration was determined by immunobloting. The DnaA protein amount per cell was then estimated through counting the number of cells used in the measurement.

The DnaA protein exerts a positive regulation on initiation of replication [32], [33]. The amount and/or concentration of DnaA protein are thought to be a limiting factor for initiation of replication. It could be possible that AspC affects initiation of replication by changing the amount or concentration of DnaA. To test this posibility we determined the amount of DnaA protein per wild-type and Δ*aspC* cells, both with and without an extra supply of AspC. Cell extracts from exponentially growing cells in ABTGcasa medium at 37°C were prepared and the total amount of protein per cell was measured as described in Materials and Methods. Also the DnaA concentration in these cell extracts were measured by Western blotting and observed not to be changed in the absence of AspC or presence of extra AspC, relative to wild type (Figure 6A). Further comparison and calculation showed that the DnaA amount per cell was increased in the presence of excess AspC but decreased in the absence of AspC, relative to wild type (Figure 6B). The results suggest that AspC affects initiation by changing the amount of DnaA protein per cell and not by altering the concentration of DnaA.

### AspC does not Change the Temperature Sensitive Phenotype of the *dnaA*46, *dnaB*252 and *dnaC*2 Mutants

As mentioned in the introduction, DnaA, DnaB and DnaC are essential components for initiation of replication and DnaB is also required for elongation of replication as a helicase. The mutant proteins DnaA46, DnaB252 and DnaC2 are thermosensitive but functional at permissive temperature (30°C). However, they are inactivated at the non-permissive temperature (42°C) and the cells die as a result of blockage of replication [34]–[36]. To see if AspC affects initiation of replication by interacting with DnaA, DnaB or DnaC, we transferred the *aspC::kan* ^R^ allele to *dnaA*46, *dnaB*252 and *dnaC*2 mutants and tested the temperature sensitivity of these resulting double mutants. The results show that absence of AspC did not change the temperature sensitivity of the *dnaA*46, *dnaB*252 and *dnaC*2 mutants (Table S2 in File S1). Also overproduction of AspC by pACYC177-*aspC* was not found to change the *ts* phenotype of the mutants (Table S2 in File S1). The results indicate that AspC does not genetically interact with DnaA, DnaB and DnaC. Therefore, it is likely that AspC does not affect initiation of replication through interacting with these initiation factors.

### Decreased Number of Origins per Cell in the Absence of AspC is not due to (p)ppGpp Metabolism

**Figure 7:**
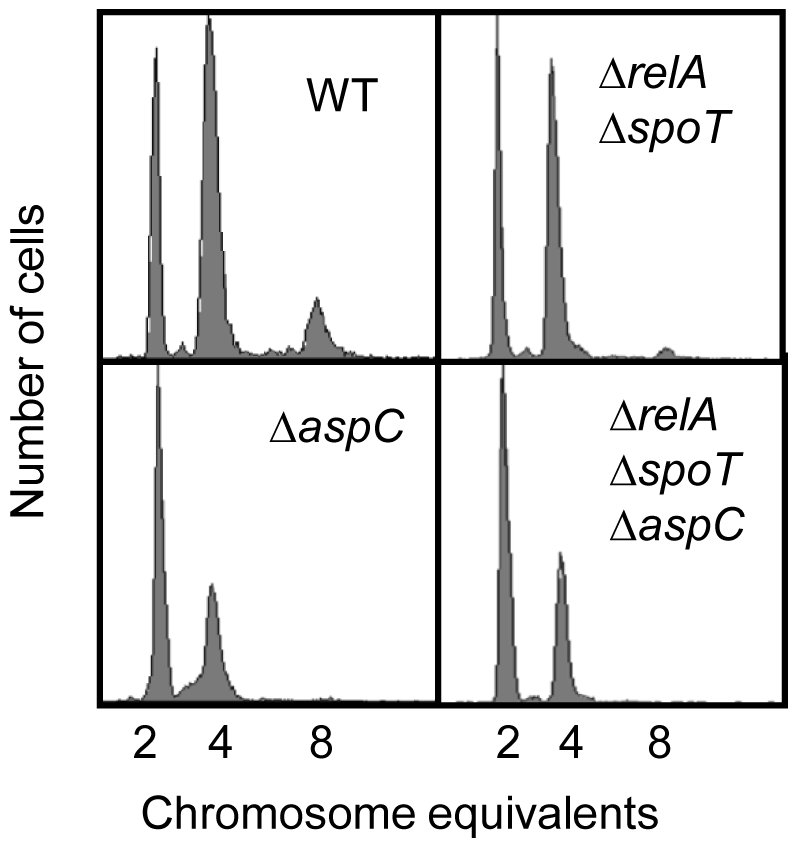
The AspC-effect on initiation of replication is unlikely to be through production of (p)ppGpp. Exponentially growing cells in ABTGcasa at 37°C were treated with rifampicin and cephalexin for 3–5 generations and then analyzed by flow cytometry as described in the legend to Figure 1.

Aspartate is a precursor of methionine and isoleucine and therefore the Δ*aspC* mutant could be starved for amino acids. A small molecule, (p)ppGpp (Guanosine pentaphosphate or tetraphosphate) synthesized by RelA and SpoT [37], is accumulated when amino acid starvation occurs [37]. It has been shown that (p)ppGpp controlled growth rate of cells [38], [39], inhibits expression of *dnaA* and the activity of DnaG by unknown processes [40], [41]. We wondered if the delay in initiation of replication in the absence of AspC was caused by production of (p)ppGpp. To test this hypothesis we transferred the *aspC::kan* ^R^ allele to MG1655Δ*relA*Δ*spoT* mutant, which is deficient in production of (p)ppGpp, resulting in the triple mutant Δ*aspC*Δ*relA*Δ*spoT*. Exponentially growing cells in ABTGcasa at 37°C were treated with rifampicin and cephalexin for 3–5 generations, and then analyzed by flow cytometry. Compared to wild type cells, Δ*relA*Δ*spoT* had less 8-origin and slightly more 2-origin cells (Figure 7) and a slower growth rate (doubling time was 46 min relative to 34 min in wild-type cells)(Table S3 in File S1). However, the triple mutant Δ*aspC*Δ*relA*Δ*spoT* showed the same replication pattern as *aspC::kan* ^R^ (Figure 7), indicating that the effect of AspC on initiation is not caused by production of (p)ppGpp. It is worth mentioning that growth of the triple mutant was slowed down without a decrease in cell size (Table S3 in File S1) relative to Δ*aspC* and Δ*relA*Δ*spoT* cells. (p)ppGpp is required for effective transcription of genes encoding enzymes for amino acid biosynthesis [37] and depletion of (p)ppGpp results in an auxotrophic phenotype for some amino acids [37]. The lack of amino acids may explain why replication initiation is delayed in the Δ*relA*Δ*spoT* mutant.

### AspC is Structurally Conserved in Evolution

To investigate if AspC-mediated coordination of the cell cycle in *E. coli* could apply to other organisms we compared the protein sequence of AspC (or homologues) from gram-negative bacteria (*Salmonella enteric, Citrobacter rodentium*), gram-positive bacteria (*Bacillus subtilis, Mycobacterium tuberculosis*), yeast (*Saccharomyces cerevisiase*), archae (*Sulfolobus solfataricus, S. acidocaldarius*), plant (*Arabidopsis thaliana*) and mammals (*Cricetulus griseus, Homo sapiens*) with that of the *E. coli* AspC (Figure S5). We found that 47.5% of the AspC sequences (or homologues) of the organisms mentioned above were identical, indicating that AspC/aspartate aminotransferase is structurally conserved in bacteria, archae, yeast, plant and mammals (Figure S5).

To investigate whether a supplement of aspartate also would affect the cell cycle of other organsims, we determined the growth rate and cell size of exponentially growing *Salmonella enteric* in the ABTG medium at 37°C. We found that addition of aspartate led to production of bigger cells with faster growth (Figure S6).

## Discussion

### AspC-mediated Aspartate Metabolism Affects Progression of the Cell Cycle

The cell cycle in fast-growing *E. coli* consists of at least two independent cycles, i.e., a chromosome replication and a cell division cycle [42]. However, it is not clear how the replication and division cycles are coupled. The nutrient availability and metabolic status are suggested to coordinate cell growth with chromosome replication and cell division [2]. In support of this idea, a richer medium leads to a decrease in mass doubling time and an increase in cell size. Several genetic analyses show that Pgk, Pgm, Eno, PykA [13], Pta and AckA [10] proteins, which are involved in sugar metabolism, are also associated with DNA replication by unknown factors. A recent study shows that the FabH protein-mediated fatty acid biosynthesis plays a central role in regulating the size of *E. coli* cells in response to nutrient availability [14]. Small signalling molecules (p)ppGpp may provide links between the cell cycle and the general nutritional status of the cell [2].

However, it is not yet clear if there exists any regulator that affects both the replication and division cycles through a metabolic pathway. In the present study, we show that aspartate metabolism affects the *E. coli* cell cycle. Deletion of the *aspC* gene leads to generation of small cells with less replication origins and a prolonged doubling time. Excess AspC exerts the opposite effect. The function of AspC-mediated aspartate metabolism in the control of the cell cycle is observed to be specific, since only extra aspartate of the 20 amino acids increase the number of origins per cell and cell size simultaneously (Figure 5, S3 and S4). Although additional glutamate increases the number of origins per cell in wild type, all the other 18 amino acids do not (Figure S3). Furthermore, glutamate does not increase the cell size as aspartate does (Table S1 in File S1). Hence, we propose that AspC-mediated metabolism of aspartate is likely to be a coordinating mechanism of the *E. coli* cell cycle.

### AspC is a Key Protein Connecting Amino Acids, Sugar and Fatty Acid Metabolism

To the extent studied, AspC does not affect the cell cycle through interacting with replication proteins or by promoting production of the small signalling molecule (p)ppGpp. We consider that AspC-mediated aspartate metabolism functions as a coordinating mechanism of the *E. coli* cell cycle, as it is an important metabolic pathway connecting sugar,amino acid and fatty acid synthesis. It is known that L-aspartate is involved in the biosynthesis of multifarious substances including amino acids, mDAP (meso-diaminopimelate), purine and pyrimidine nucleotides, NAD+, and pantothenic acid [43]–[48]. CoA, an important coenzyme derived from pantothenic acid, is responsible for amino acid metabolism, tricarboxylic acid cycle (TCA cycle) and fatty acid synthesis [49]. AspA converts aspartate to fumarate, which is a component of the TCA cycle. Fumarate and aspartate are able to activate the DcuS/DcuR two-component system which controls the C4-dicarboxylate utilization [50]. Also phosphorylated NarL, the regulator of NarX/NarL two-component system which senses the level of nitrate, suppresses the expression of AspA indirectly [50]. Hence, it regulates the interconversion of aspartate and fumarate. We therefore think it is likely that the aspartate metabolism may be a key process that connects amino acid, sugar and fatty acid metabolism with DNA replication, cell growth and cell division.

### The AspC-mediated Aspartate Metabolism Affects the Amount of DnaA per Cell

**Figure 8:**
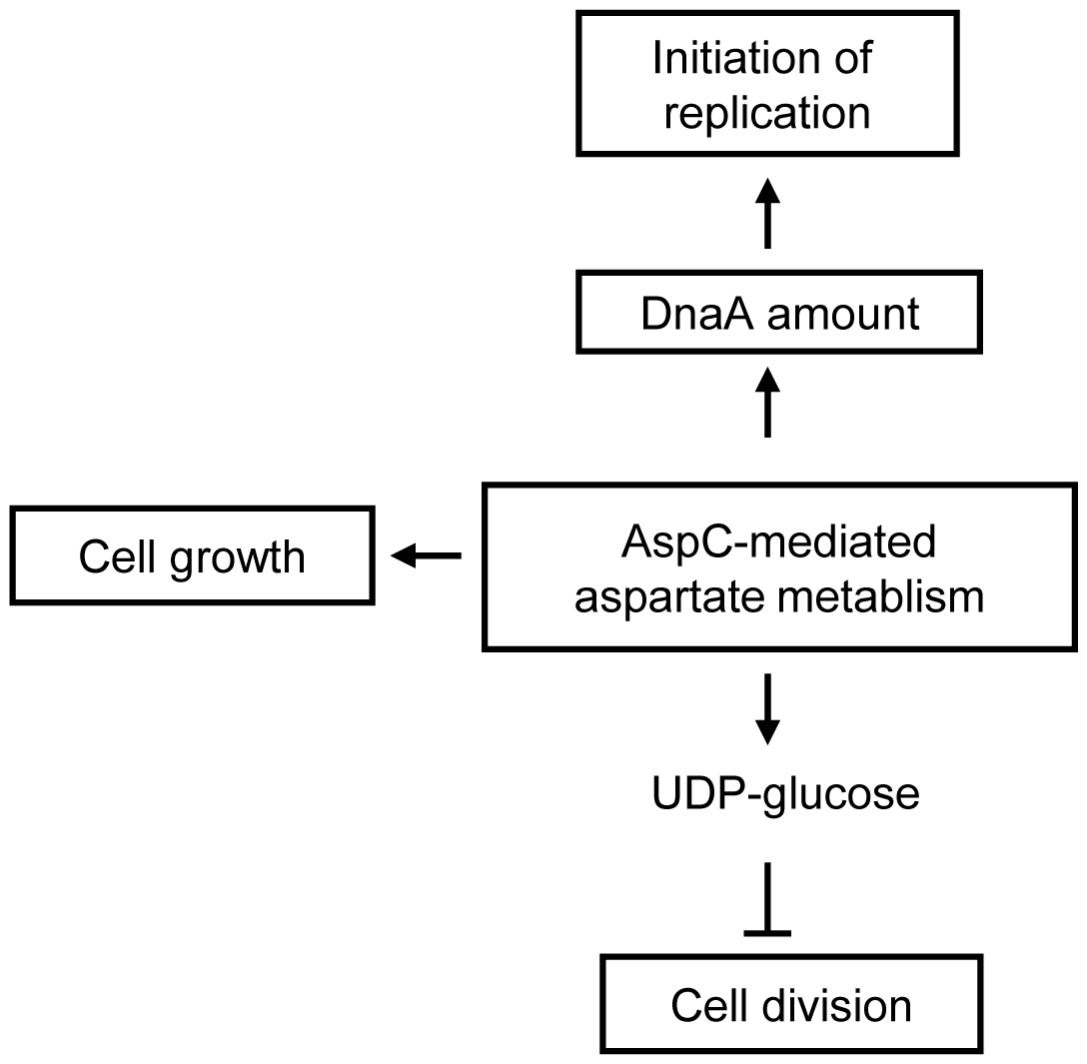
A model of AspC-mediated coordination of cell cycle. The AspC-mediated aspartate metabolism stimulates protein synthesis, which subsequently increases the amount of DnaA per cell and triggers initiation of replication. AspC-mediated aspartate metabolism delays cell division by accumulating UDP-glucose but activates cell growth. The model proposes that AspC-mediated aspartate metabolism coordinates cell growth with the replication and the division cycle.

Nutrient availability has a dramatic effect on accumulation of DnaA [2]. It is suggested that DnaA accumulates in rich medium and hence, triggers replication initiation [51]. Conversely, cells starving for amino acids stops *de novo* DnaA synthesis and subsequently prevents initiation of replication [52]. In accordance with these observations, we show here that deletion or overproduction of AspC leads to a decrease or an increase in the amount of DnaA per cell, respectively, but does not change the temperature sensitivity of *dnaA46*, *dnaB252* and *dnaC2*. The results suggest that AspC-mediated aspartate metabolism may indirectly regulate the initiation frequency of replication through changing the amount of DnaA per cell (Figure 8). A similar way of DnaA function on initiation of replication was shown in Δ*pgm* and *ftsA* mutants [17]. Elongation of replication is not influenced by AspC (Figure S7), indicating that AspC-mediated aspartate metabolism most likely does not contribute to elongation of replication.

### Growth Rate is not Always a Measure for Initiation of Replication and Cell Division

Carbon availability determines cell size for fast growing bacteria [53], [54] and the message of carbon availability is directly delivered to the division apparatus through UDP-glucose accumulation [55]. UDP-glucose inhibits cell division by its interaction with bifunctional diacylglycerol glucosyltransferase, UgtP in *Bacillus subtilis* [55]. OpgH, the integral inner-membrane protein, inhibits FtsZ ring formation in an UDP-glucose dependent manner in *E. coli* [56]. It is obvious that the *E. coli* OpgH is a functional homologue of the *B. subtilis* UgtP. During fast growth in rich medium, UDP-glucose is accumulated, resulting in inhibition of FtsZ assembly by UgtP or OpgH and a subsequent delay of cell division until cells have reached the appropriate length. Conversely, under slow growth condition in poor medium, the cells divide at a shorter length due to lowered amount of UDP-glucose [2]. The *E. coli* phosphoglucomutase (Pgm) and glucose-1-phosphate uridylytransfease (GalU) seem to be required for UDP-glucose biosynthesis and mutations in *pgm* and *galU* genes reduce the *E. coli* cell size by 30% [17], [57]. The amount of UDP-glucose per cell is not determined in this work, but it is reasonable to assume that aspartate metabolism-dependent alterations in cell size are due to fluctuations in the amount of UDP-glucose (Figure 8).

Cell growth depends on nutrient availability. Rich media lead to an increase in both growth rate and cell size whereas poor media reduces growth rate and cell size [53], [58]. Alterations in growth rate is accompanied with changes in timing of replication initiation and cell division [2], which can be measured as changes in number of origins per cell and cell size. Thus, nutrient availability is crucial for timing of cell cycle progression and thereby growth rate. When cells are growing fast, the number of origins per cell is found to be high in big cells; conversely, the number of origins per cell is low in small cells when cells are growing slowly. However, the number of origins per cell, the initiation mass and cell size are not always linear under nutrient dependent growth (Figure 1B, 2B and S2). As shown previously, DnaA determines the initiation mass [33]. It is therefore likely that aspartate metabolism-dependent amounts of DnaA and UDP-glucose determine timing of replication initiation and cell division in a coordinated manner (Figure 8).

## Supporting Information

Figure S1 The connections between carbon metabolism and amino acid synthesis in *E. coli*. The Pgk, Gpm, Eno, and PykA protein, which are known to participate in carbon metabolism, are suggested to be involved in regulation of replication elongation, while AckA and Pta have been shown to be involved in replication initiation. AspC catalyzes synthesis of Asp, Phe, and Tyr. Other metabolic connections between sugar metabolism and amino acid synthesis are also shown.
(TIF)

Figure S2 Correlation between the initiation mass and growth rate in Δ*aspC* mutant. To determine the initiation age (*a_i_*) and the number of origins per cell at initiation (*O_i_*), exponentially growing cells at 37°C in ABTG, ABTGcasa and LB medium (see Materials and Methods) were treated with rifampicin and cephalexin for 3–5 generations and analyzed by flow cytometry as described in the Materials and Methods. Exponentially growing cells in the media mentioned above were also harvested to determine average cell mass (*M_av_*) by a fluorescent microscopy. Then the initiation mass (*M_i_*) was calculated from the expression *M_i_* = M_av_*2*^ai^*/2ln*O_i_ as described previously (Wold et al., 1994). The relative initiation masses measured (Y-axis) were plotted as a function of number of doublings per hour (X-axis). The dashed arrows indicate the values measured in the medium indicated. The values are average of three individual experiments, standard errors are given.
(TIF)

Figure S3 Addition of aspartate or glutamate increases while cysteine, alanine or leucine decreases the number of origins per cell in wild-type cell. Exponentially growing wild-type cells at 37°C in ABTG medium (see Materials and Methods) supplemented with amino acids as noted at 100 μg/mL were treated with rifampicin and cephalexin for 3–5 generations and analyzed by flow cytometry as described in Materials and Methods. For each analysis, 10000 cells were included. The amino acid added (or not) in the medium is indicated in each panel.
(TIF)

Figure S4 Supplementation of aspartate recovers a wild-type replication pattern in Δ*aspC* cells. Exponentially growing Δ*aspC* cells at 37°C in ABT medium (see Materials and Methods) supplemented with amino acid as noted at 100 μg/mL were collected by centrifugation, and analyzed by flow cytometry as described in Materials and Methods. For each analysis, 10000 cells were included. The amino acid added (or not) in the medium is indicated in each panel.
(TIF)

Figure S5 AspC is conserved in both prokaryotes and eukaryotes. The protein sequence of AspC (Aspartate aminotransferase) from gram-negative bacteria (*Escherichia coli, Salmonella enteric* and *Citrobacter rodentium*), gram-positive bacteria (*Bacillus subtilis* and *Mycobacterium tuberculosis*), plant (*Arabidopsis thaliana*), Yeast (*Saccharomyces cerevisiae*), mammals (*Cricetulus griseus* and *Homo sapiens*) and Archae (*Sulfolobus solfataricus* and *Sulfolobus acidocaldarius*) were aligned and analyzed. The protein sequences were derived from the web site: http://www.uniprot.org/.
(TIF)

Figure S6 Cell length of wild-type *Salmonella enteric* is increased, with faster growth in the presence of extra aspartate. Exponentially growing wild-type cells at 37 in ABTG medium (see Materials and Methods) supplemented with amino acids as noted at 100 μg/mL were harvested, fixed in 70% ethanol, and then cell sizes were measured using microscopy. Each experiment included about 100 cells. Doubling time for wild type cells was 45 min in the absence of aspartate and 39 min, 42 min or 47 min in the presence of aspartate, arginine or alanine, respectively. The amino acid added in the medium is indicated.
(TIF)

Figure S7 Chain elongation rate is not changed in the absence of AspC or presence of excess AspC. Exponentially growing cells at 37°C in ABTGcasa medium (see Materials and Methods) were treated with rifampicin and cephalexin, then harvested by centrifugation at 0, 15, 30, 45, 60, 75 and 90 minutes after rifampicin and cephalexin treatment. Cells were fixed in 70% ethanol and analyzed by flow cytometry. For each analysis, 10000 cells were included. The time (min) of rifampicin and cephalexin treatment is indicated (top) and the strains tested (right). To measure chain elongation rate, we compared changes in the DNA histograms of cells taken at the time intervals indicated after addition of rifampicin and cephalexin. The kinetics of this change reflects the rate of replication fork movement (Morigen *et al*., 2003). Complete sharp peaks of cells with 2, 4, 8-chromosomes appeared after 90 min in both wild type, Δ*aspC* cells and cells with excess AspC, indicating that chain elongation proceeds at the same rate in the four different strains. The results suggest that chain elongation rate is not dependent on AspC.
(TIF)

File S1 **Table S1**, Cell cycle parameters of wild type cells in ABTG medium with amino acids. **Table S2**, Deletion or overproduction of AspC does not change the temperature sensitivity of *dnaA46*, *dnaB252* and *dnaC2*. **Table S3,** The delay of the cell cycle in Δ*aspC* is unlikely to be caused by (p)ppGpp.
(DOC)
