# Supplementary figures and images for: AspC-Mediated Aspartate Metabolism Coordinates the Escherichia coli Cell Cycle
Source: PLoS One. 2014 Mar 26;9(3):e92229. doi: 10.1371/journal.pone.0092229 (PMC3966765; doi:10.1371/journal.pone.0092229)

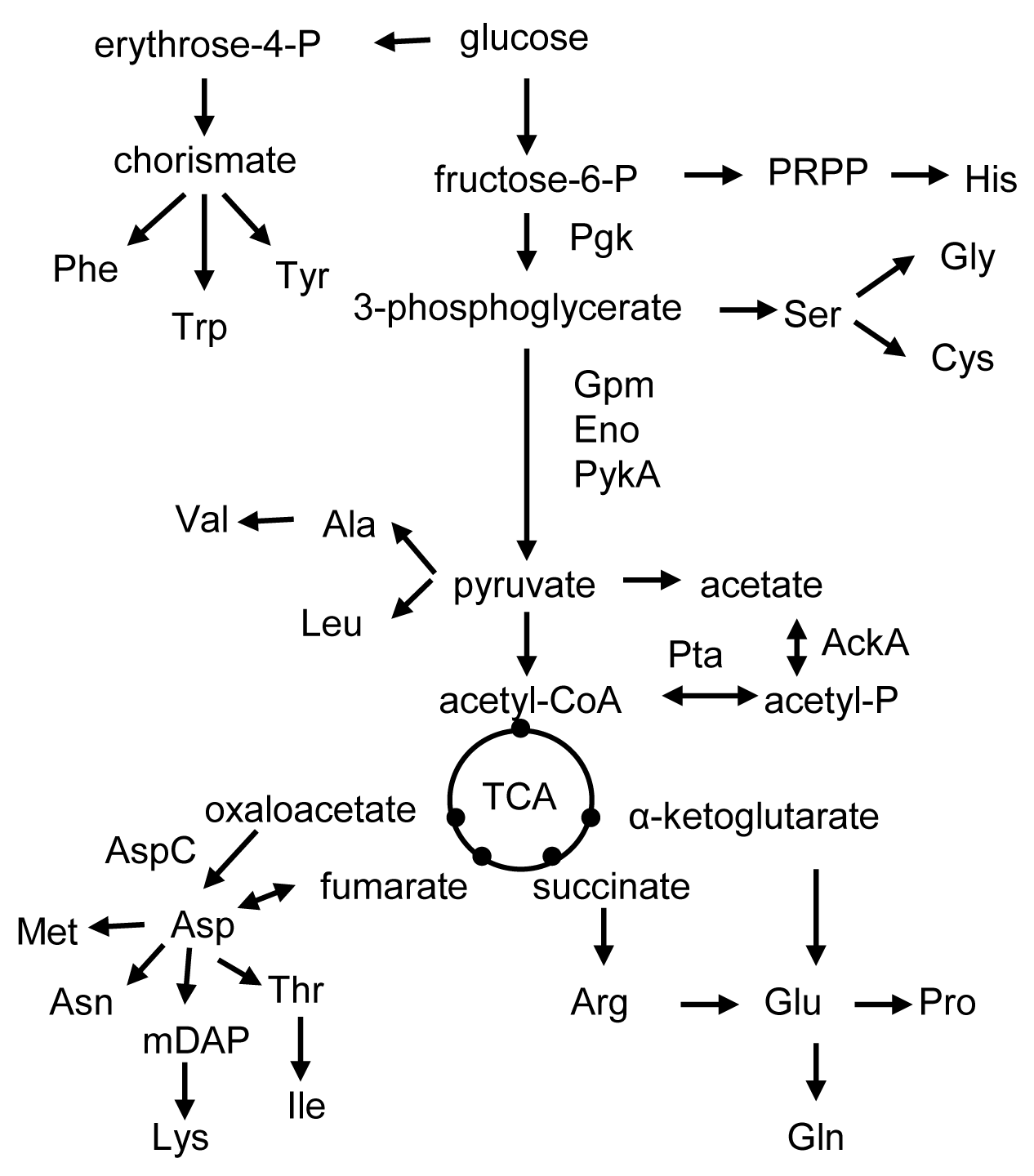

Supplement: Figure S1 — The connections between carbon metabolism and amino acid synthesis in E. coli. The Pgk, Gpm, Eno, and PykA protein, which are known to participate in carbon metabolism, are suggested to be involved in regulation of replication elongation, while AckA and Pta have been shown to be involved in replication initiation. AspC catalyzes synthesis of Asp, Phe, and Tyr. Other metabolic connections between sugar metabolism and amino acid synthesis are also shown. (TIF) [file pone.0092229.s001.tif]

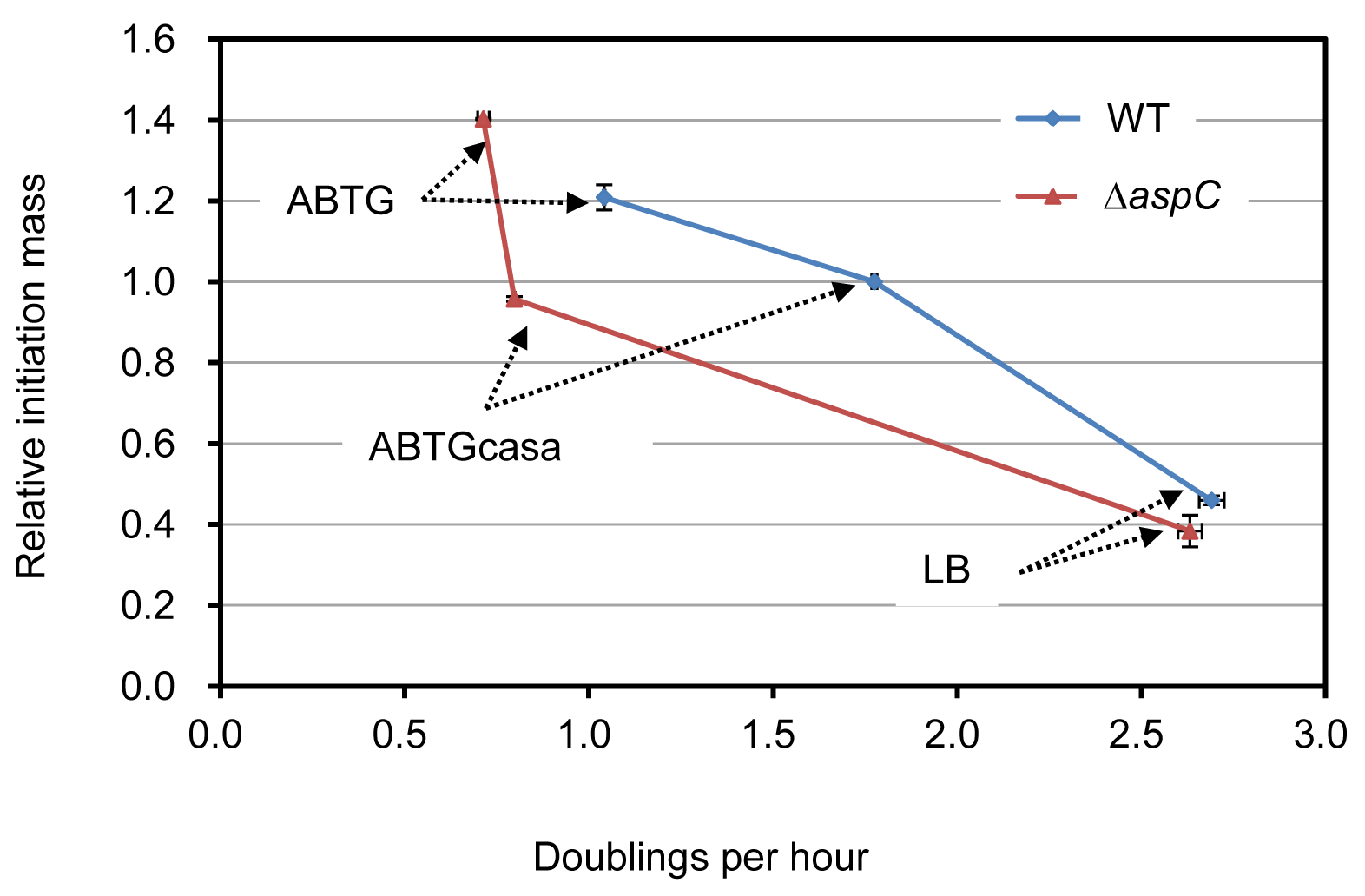

Supplement: Figure S2 — Correlation between the initiation mass and growth rate in ΔaspC mutant. To determine the initiation age (ai) and the number of origins per cell at initiation (Oi), exponentially growing cells at 37°C in ABTG, ABTGcasa and LB medium (see Materials and Methods) were treated with rifampicin and cephalexin for 3–5 generations and analyzed by flow cytometry as described in the Materials and Methods. Exponentially growing cells in the media mentioned above were also harvested to determine average cell mass (Mav) by a fluorescent microscopy. Then the initiation mass (Mi) was calculated from the expression Mi = Mav*2ai/2ln*Oi as described previously (Wold et al., 1994). The relative initiation masses measured (Y-axis) were plotted as a function of number of doublings per hour (X-axis). The dashed arrows indicate the values measured in the medium indicated. The values are average of three individual experiments, standard errors are given. (TIF) [file pone.0092229.s002.tif]

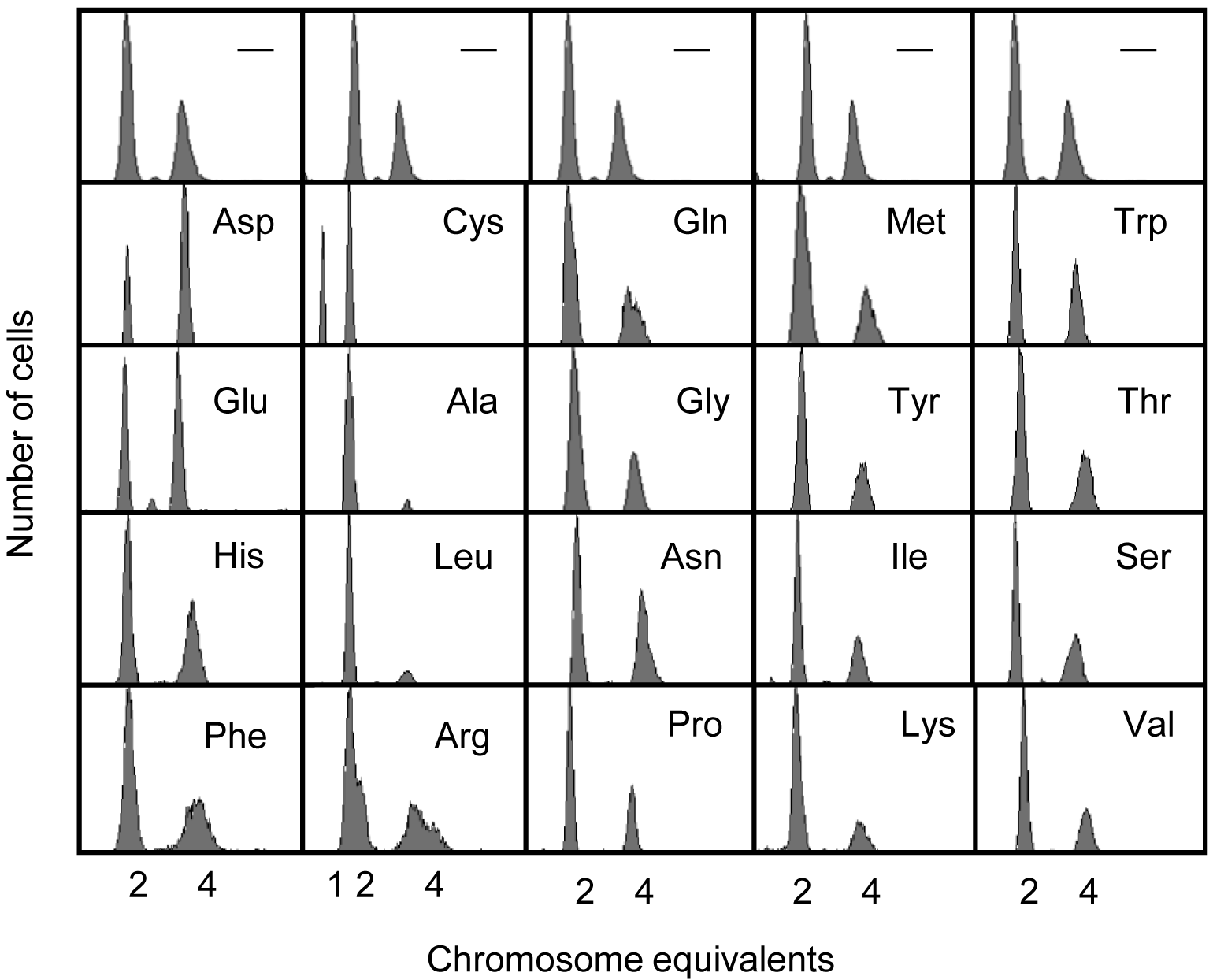

Supplement: Figure S3 — Addition of aspartate or glutamate increases while cysteine, alanine or leucine decreases the number of origins per cell in wild-type cell. Exponentially growing wild-type cells at 37°C in ABTG medium (see Materials and Methods) supplemented with amino acids as noted at 100 μg/mL were treated with rifampicin and cephalexin for 3–5 generations and analyzed by flow cytometry as described in Materials and Methods. For each analysis, 10000 cells were included. The amino acid added (or not) in the medium is indicated in each panel. (TIF) [file pone.0092229.s003.tif]

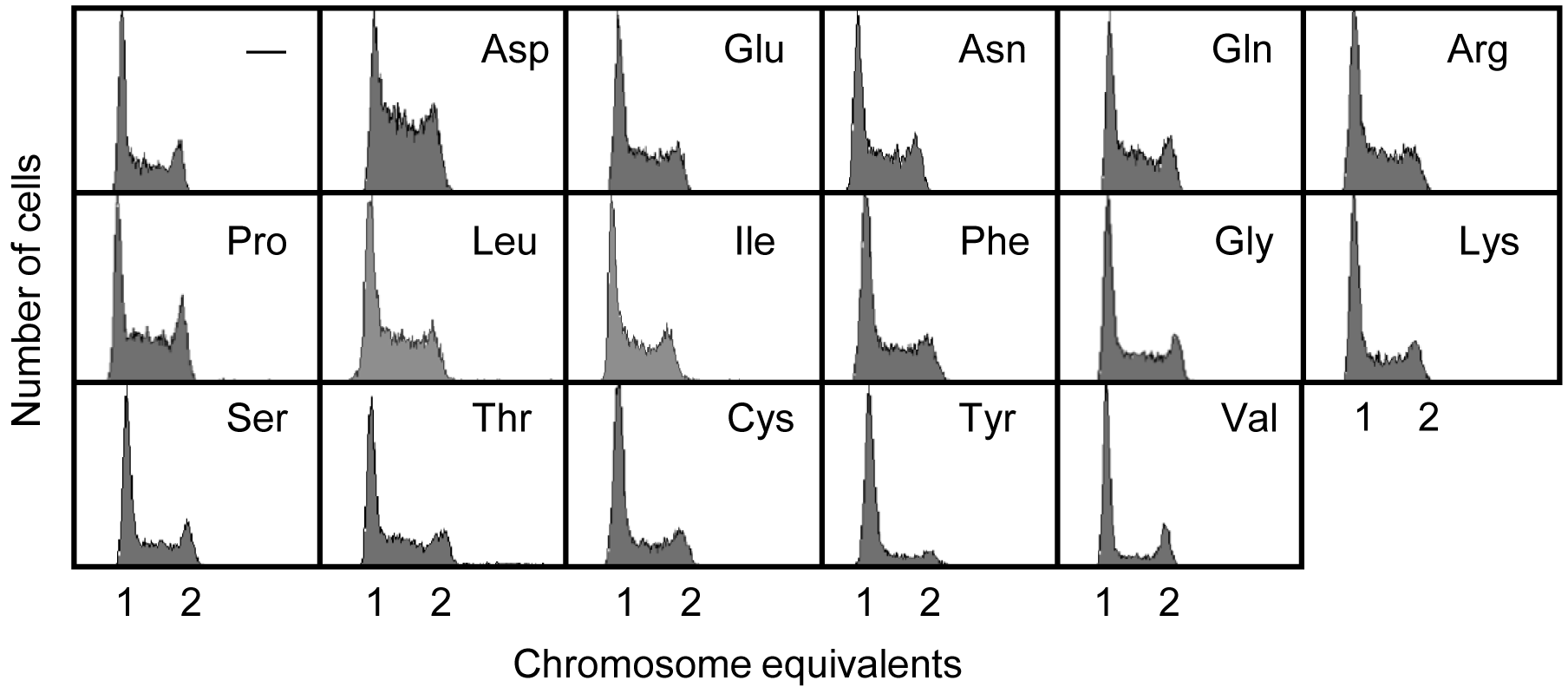

Supplement: Figure S4 — Supplementation of aspartate recovers a wild-type replication pattern in ΔaspC cells. Exponentially growing ΔaspC cells at 37°C in ABT medium (see Materials and Methods) supplemented with amino acid as noted at 100 μg/mL were collected by centrifugation, and analyzed by flow cytometry as described in Materials and Methods. For each analysis, 10000 cells were included. The amino acid added (or not) in the medium is indicated in each panel. (TIF) [file pone.0092229.s004.tif]

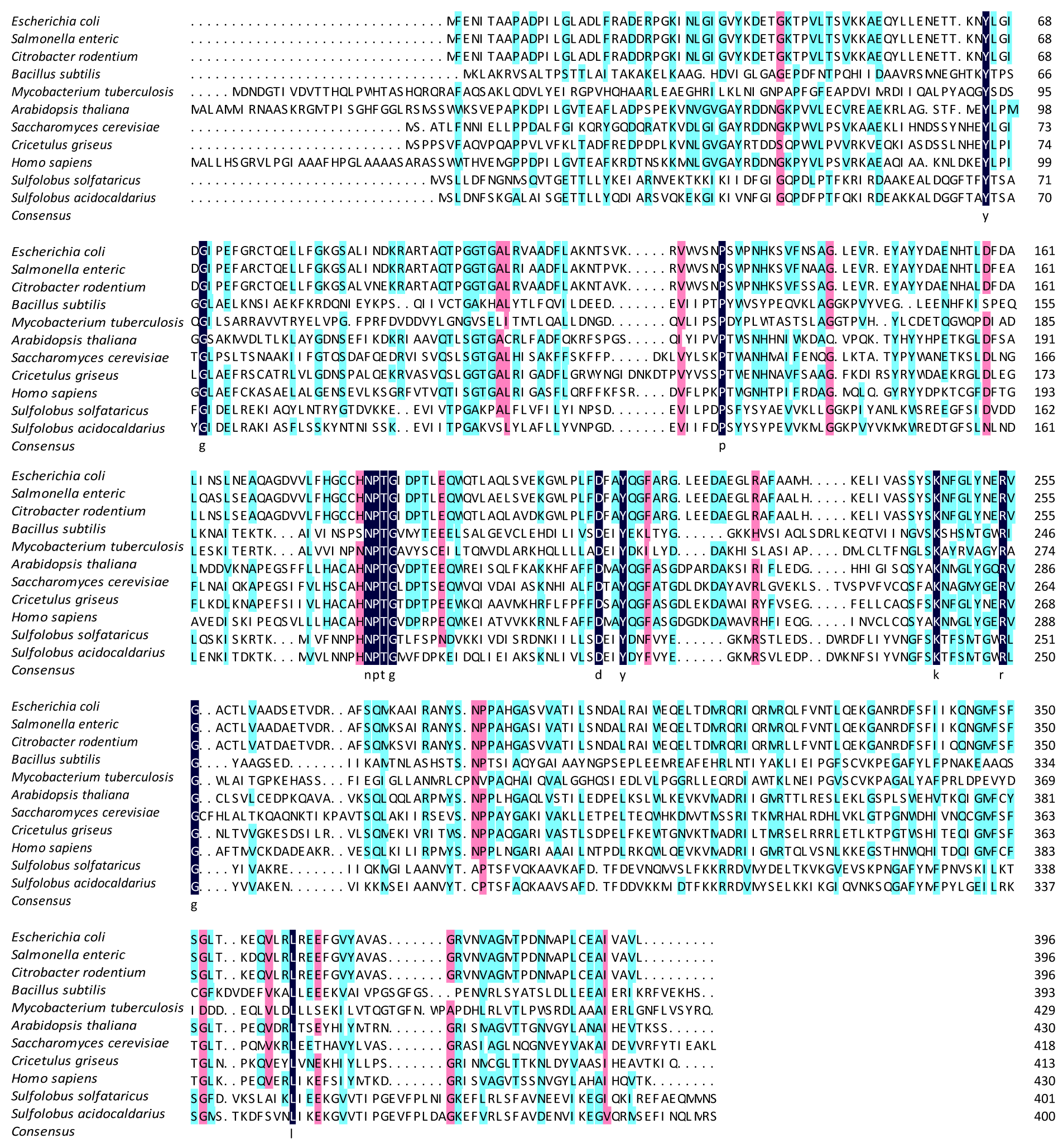

Supplement: Figure S5 — AspC is conserved in both prokaryotes and eukaryotes. The protein sequence of AspC (Aspartate aminotransferase) from gram-negative bacteria (Escherichia coli, Salmonella enteric and Citrobacter rodentium), gram-positive bacteria (Bacillus subtilis and Mycobacterium tuberculosis), plant (Arabidopsis thaliana), Yeast (Saccharomyces cerevisiae), mammals (Cricetulus griseus and Homo sapiens) and Archae (Sulfolobus solfataricus and Sulfolobus acidocaldarius) were aligned and analyzed. The protein sequences were derived from the web site: http://www.uniprot.org/. (TIF) [file pone.0092229.s005.tif]

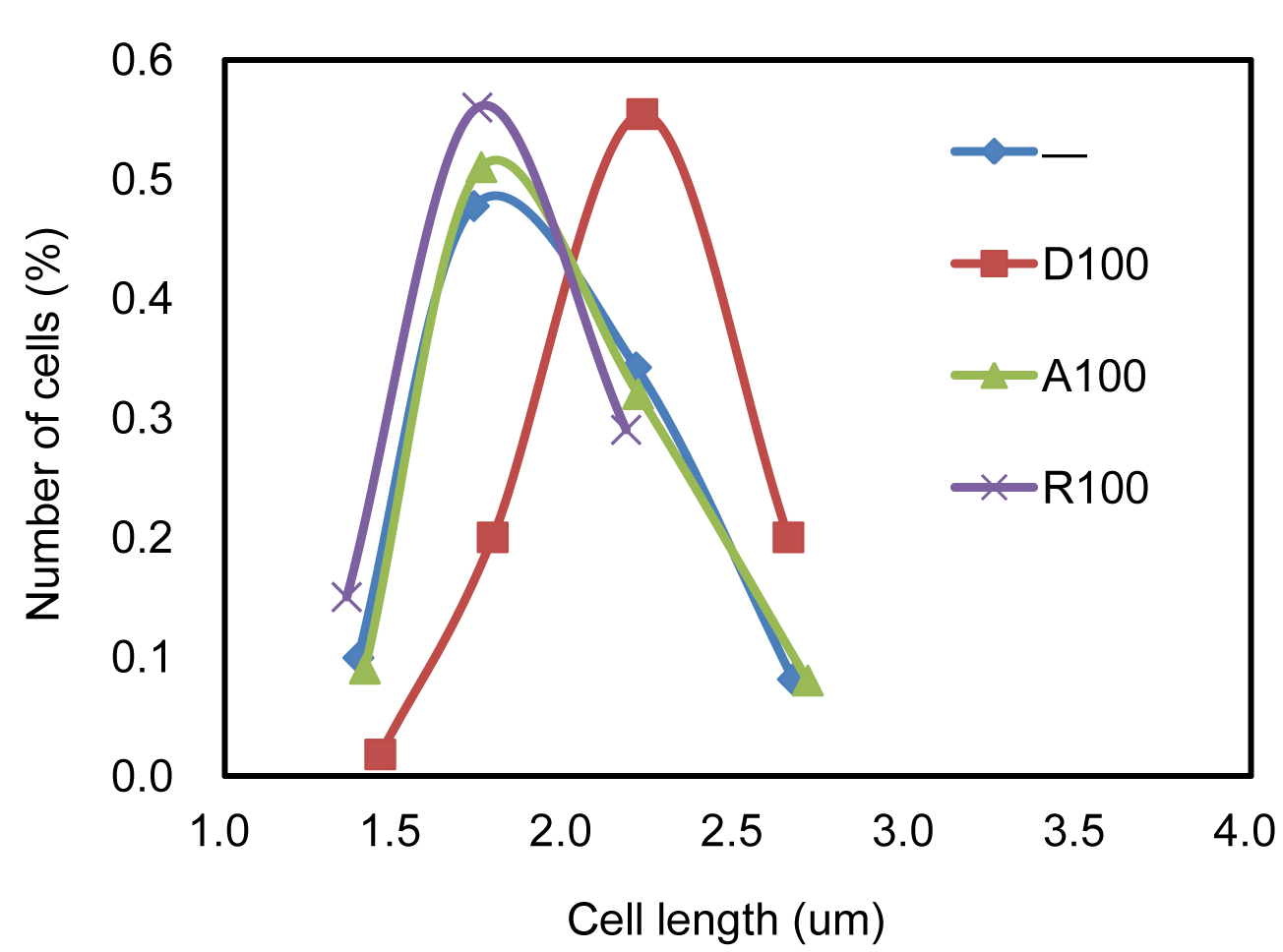

Supplement: Figure S6 — Cell length of wild-type Salmonella enteric is increased, with faster growth in the presence of extra aspartate. Exponentially growing wild-type cells at 37 in ABTG medium (see Materials and Methods) supplemented with amino acids as noted at 100 μg/mL were harvested, fixed in 70% ethanol, and then cell sizes were measured using microscopy. Each experiment included about 100 cells. Doubling time for wild type cells was 45 min in the absence of aspartate and 39 min, 42 min or 47 min in the presence of aspartate, arginine or alanine, respectively. The amino acid added in the medium is indicated. (TIF) [file pone.0092229.s006.tif]

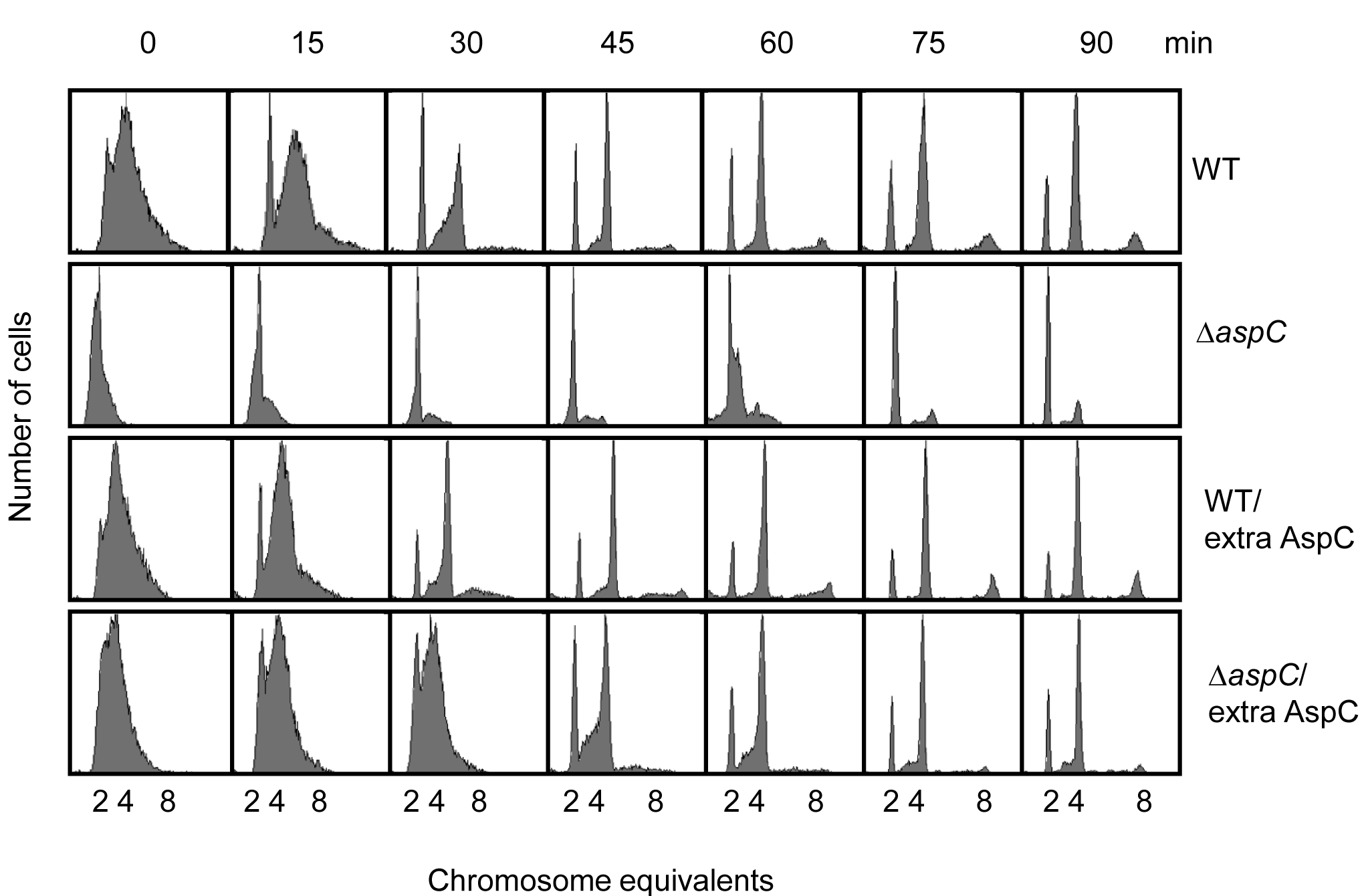

Supplement: Figure S7 — Chain elongation rate is not changed in the absence of AspC or presence of excess AspC. Exponentially growing cells at 37°C in ABTGcasa medium (see Materials and Methods) were treated with rifampicin and cephalexin, then harvested by centrifugation at 0, 15, 30, 45, 60, 75 and 90 minutes after rifampicin and cephalexin treatment. Cells were fixed in 70% ethanol and analyzed by flow cytometry. For each analysis, 10000 cells were included. The time (min) of rifampicin and cephalexin treatment is indicated (top) and the strains tested (right). To measure chain elongation rate, we compared changes in the DNA histograms of cells taken at the time intervals indicated after addition of rifampicin and cephalexin. The kinetics of this change reflects the rate of replication fork movement (Morigen et al., 2003). Complete sharp peaks of cells with 2, 4, 8-chromosomes appeared after 90 min in both wild type, ΔaspC cells and cells with excess AspC, indicating that chain elongation proceeds at the same rate in the four different strains. The results suggest that chain elongation rate is not dependent on AspC. (TIF) [file pone.0092229.s007.tif]
