# Supplementary material for: AspC-Mediated Aspartate Metabolism Coordinates the Escherichia coli Cell Cycle
Source: PLoS One. 2014 Mar 26;9(3):e92229. doi: 10.1371/journal.pone.0092229 (PMC3966765; doi:10.1371/journal.pone.0092229)
Supplement: File S1 — Table S1, Cell cycle parameters of wild type cells in ABTG medium with amino acids. Table S2, Deletion or overproduction of AspC does not change the temperature sensitivity of dnaA46, dnaB252 and dnaC2. Table S3, The delay of the cell cycle in ΔaspC is unlikely to be caused by (p)ppGpp. (DOC) [file pone.0092229.s008.doc]

Table S1 Cell cycle parameters of wild type cells in ABTG medium with amino acids.

| a.a. | A.O.^a^ | Cell size(μm)^b^ | Doubling time(min) |
| --- | --- | --- | --- |
| - | 2.43±0.19 | 2.32±0.01 | 57.6±0.1 |
| Asp | 3.10±0.16 | 2.45±0.07 | 49.1±2.2 |
| Gly | 2.53±0.01 | 2.14±0.40 | 52.2±0.6 |
| Tyr | 2.78±0.30 | 2.35±0.36 | 49.3±1.9 |
| Ala | 2.19±0.02 | 2.04±0.42 | 57.7±1.9 |
| His | 2.63±0.02 | 2.02±0.37 | 61.2±3.7 |
| Met | 2.78±0.07 | 2.14±0.33 | 51.4±1.7 |
| Trp | 2.71±0.05 | 2.26±0.37 | 56.8±2.5 |
| Glu | 2.84±0.14 | 2.31±0.06 | 51.5±2.0 |
| Pro | 2.56±0.06 | 2.24±0.12 | 55.1±1.8 |
| Asn | 2.78±0.14 | 2.11±0.35 | 51.2±0.6 |
| Leu | 2.20±0.01 | 2.08±0.34 | 67.5±2.5 |
| Ile | 2.43±0.16 | 2.14±0.32 | 56.4±0.7 |
| Cys | 1.33±0.07 | 1.74±0.39 | 256.3±2.9 |
| Ser | 2.61±0.02 | 2.29±0.34 | 57.3±2.6 |
| Gln | 2.73±0.15 | 2.31±0.13 | 50.5±2.6 |
| Val | 2.55±0.08 | 2.14±0.32 | 60.5±6.2 |
| Arg | 2.60±0.08 | 2.26±0.18 | 53.0±2.9 |
| Phe | 2.58±0.04 | 2.21±0.33 | 54.4±0.9 |
| Lys | 2.46±0.03 | 2.10±0.35 | 56.7±2.3 |
| Thr | 2.81±0.06 | 2.22±0.35 | 54.6±1.3 |

a Exponentially growing wild type cells at 37°C in ABTG medium supplemented with amino acids (as noted) at 100μg/mL were treated with rifampicin and cephalexin for 3-5 generations and analyzed by flow cytometry. The average number of origins per cell (A. O.) was calculated using the flow cytometry data obtained.

b Exponentially growing cells as mentioned above were harvested, fixed in 70% ethanol and then cell sizes were measured using microscopy. Each experiment included more than 100 cells. The values are average of three individual experiments, standard errors are shown.

Table S2 Deletion or overproduction of AspC does not change the temperature sensitivity of *dnaA46*, *dnaB252* and *dnaC2*.

The *aspC::kan*^R^ allele was transferred to *dnaA*46, *dnaB*252 and *dnaC*2 mutants by P1 transduction. The transductants were restreaked on LB agar plates with required antibiotics and then the survival ratio was tested by culturing the cells at 30°C, 37°C and 42℃. Extra AspC was produced from pACYC177-*aspC* plasmid as described in Materials and Methods.

| strain | genotype | 30°C | 37°C | 42°C |
| --- | --- | --- | --- | --- |
| MOR687 | *dnaA46(Ts)* | 8/8 | 8/8 | 0/8 |
| MOR823 | *dnaA46(Ts) aspC::Kan*^R^ | 8/8 | 8/8 | 0/8 |
| MOR832 | *dnaA46(Ts)*/extra AspC | 8/8 | 8/8 | 0/8 |
| MOR227 | *dnaB252(Ts)* | 8/8 | 8/8 | 0/8 |
| MOR824 | *dnaB252(Ts)* *aspC::Kan*^R^ | 8/8 | 8/8 | 0/8 |
| MOR833 | *dnaB252(Ts)*/extra AspC | 8/8 | 8/8 | 0/8 |
| MOR166 | *dnaC2(Ts)* | 8/8 | 8/8 | 0/8 |
| MOR825 | *dnaC2(Ts) aspC::Kan*^R^ | 8/8 | 7/8 | 0/8 |
| MOR834 | *dnaC2(Ts)*/extra AspC | 8/8 | 4/8 | 0/8 |

Table S3 The delay of the cell cycle in *∆aspC* is unlikely to be caused by (p)ppGpp.

| strain | genotype | A.O.^a^ | Cell size(μm)^b^ | Doubling time(min) |
| --- | --- | --- | --- | --- |
| MG1655 | Wild type | 3.87±0.59 | 1.97±0.33 | 34.2±2.2 |
| MOR828 | *∆aspC* | 2.49±0.32 | 1.54±0.30 | 43.0±3.5 |
| CF1961 | *∆relA∆spoT* | 3.31±0.41 | 1.84±0.41 | 46.0±2.7 |
| MOR829 | *∆relA∆spoT∆aspC* | 2.04±0.12 | 1.48±0.29 | 65.1±1.1 |

a Exponentially growing cells at 37°C in ABTGcasa medium were treated with rifampicin and cephalexin for 3-5 generations and analyzed by flow cytometry. The average number of origins per cell (A. O.) was calculated using the flow cytometry data obtained.

b Exponentially growing cells as mentioned above were harvested, fixed in 70% ethanol and then cell sizes were measured using microscopy Each experiment included more than 100 cells. The values are average of three individual experiments, standard errors are shown.
